# 28-Day Oral Chronic Toxicity Study of *Arctigenin* in Rats

**DOI:** 10.3389/fphar.2018.01077

**Published:** 2018-09-26

**Authors:** Yu-jun Tan, Yu-shan Ren, Lei Gao, Lan-fang Li, Li-juan Cui, Bin Li, Xin Li, Jian Yang, Ming-zhi Wang, Yuan-yuan Lv, Xiao-li Xu, Jing-chun Yao, Zhong Liu, Gui-min Zhang, Jie Li

**Affiliations:** ^1^Shandong New Time Pharmaceutical Co., LTD., Lunan Pharmaceutical Group Co. Ltd., Linyi, China; ^2^National Engineering and Technology Research Center of Chirality Pharmaceutica, Lunan Pharmaceutical Group Co. Ltd., Linyi, China; ^3^State Key Laboratory of Generic Manufacture Technology of Chinese Traditional Medicine, Lunan Pharmaceutical Group Co. Ltd., Linyi, China

**Keywords:** *Arctium lappa*, *Arctigenin*, sub-chronic toxicity, safety, drug exposure

## Abstract

*Arctium lappa* (burdock) is the most popular daily edible vegetable in China and Japan because of its general health tonic effects. Previous studies focused on the beneficial role of *Arctigenin* but neglected its potential side-effects and toxicities. In the present study, the sub-chronic toxicity profile of *Arctigenin* following 28 days of consecutive exposure was investigated in rats. The results showed that during the drug exposure period, *Arctigenin*-12 mg/kg administration resulted in focal necrosis and lymphocytes infiltration of heart ventricular septal muscle cells. In the kidney cortical zone, the renal tubular epithelial cells were swollen, mineralized, and lymphocyte infiltrated. In the liver, the partial hepatocyte cytoplasm showed vacuolation and fatty changes, focal necrosis, and interstitial lymphocyte infiltration. In the rats that underwent 36 mg/kg/day administration, there was bilateral testis and epididymis atrophy. In the lung and primary bronchus, erythrocytes and edema fluid were observed. Changes of proestrus or estrus were observed in the uterus, cervix, and vagina intimal epithelial cells. Lymphocytic focal infiltration occurred in the prostate mesenchyme. The high dosage of *Arctigenin* only decreased the body weight at day 4. At the end of the recovery period, histopathological changes were irreversible, even after withdrawal of the drug for 28 days. Focal necrosis still existed in the heart ventricular septal muscle cells and hepatocytes. Lymphocyte infiltrations were observed in the heart, renal cortex, hepatocyte, and pancreas exocrine gland. Meanwhile, atrophy occurred in the testicles and pancreas. In addition, in the *Arctigenin*-12 mg/kg group, creatinine (CREA) and brain weight were both significantly increased. The toxicokinetical study demonstrated that *Arctigenin* accumulated in the organs of rats. The food consumption, hematological, and biochemical parameters were not associated with the above results. These contradictory results might result from the lesions induced by *Arctigenin*, which were not sufficiently serious to change the parameters. These results suggest that *Arctium lappa* should be consumed daily with caution because of the potential toxicity induced by *Arctigenin*. According to all results, the lowest observed adverse effect level (LOAEL) was induced by 12 mg/kg daily exposure to *Arctigenin*, and the No-observed-adverse-effect-level (NOAEL) should be lower than 12 mg/kg.

## Introduction

Mankind has relied on plants for food since our species first arose. Subsequently, we have strived for healthier edibles to avoid diseases and extend the lifespan. Increasing numbers of plants have been found and used because of their multiple beneficial functions. Traditional Chinese Medicine (TCM) is composed of herbal medicine, acupuncture and massage (Shim and Lee, 2017). Disputes between Traditional Chinese Medicine and Western medicine have lasted for several decades because the unclear mechanisms of TCM are unacceptable for the precise therapeutic strategies of Western medicine (Brand and Zhao, 2017; Fleischer et al., 2017; Yang et al., 2017). We must admit that TCM cannot accommodate modern medicine due to its multicomponent systems, and although TCM exerts positive therapeutic effects, it might result in unknown adverse effects and toxicities. When there are more ingredients, it becomes more complex to get rid of this situation, so modern Chinese medicine focuses on extracting and purifying from herbal sources and tries to explain the potential mechanisms. Undeniable, profound breakthroughs in TCM are found after years of struggling. The most famous example is the herbal extract *artemisinin*, which is widely applied in malaria therapy, and won the Nobel Prize in 2015 (Qian et al., 2017). In recent years, even in developed countries, the patronage of herbal remedies has tremendously increased because of the belief or proven efficacy and enormous economic benefits and widest applications (Molyneux and Ward, 2015; Naji et al., 2017).

Although the safety processing and long usage history of traditional Chinese medicine in disease treatment have been proven and have accumulated for centuries, potential mutagenic, toxic and even carcinogenic effects have been rarely reported (Kassie et al., 1996; Fennell et al., 2004; Ojewole and Adewunmi, 2004). In 2012, the WHO appealed to formulate relative policy and international standards for evaluating the safety and efficacy of traditional medicine. Indubitably, there has been no delay in setting up safety evaluation criteria for herbal remedies, especially for evaluating the consequence of long-term use.

The root of *Arctiumlappa L*. is used as a daily edible vegetable in Asia, especially in China, because of its general health tonic functions. For the past several years, *Arctiumlappa* (burdock) and its active component *Arctigenin* have been reported to possess multiple bioactivities *in vivo* and *in vitro* (Holetz et al., 2002; Liu et al., 2012; Hwangbo et al., 2014; Wu et al., 2014). In addition, the potential targets and mechanisms of *Arctigenin* have been known, e.g., anti-inflammatory (via inhibition of nuclear factor kappa B (NF-κB) and inducible nitric oxide synthase (iNOS) (Liu et al., 2012; Hwangbo et al., 2014; Wu et al., 2014), immunomodulatory (via inhibiting nitric oxide, interlukin-6 (IL-6) and tumor necrosis factor-α (TNF-α) production in macrophages) (Cho et al., 2004; Zhao et al., 2009; Zhu et al., 2013), amelioration of memory impairment (by suppressing microglia activation and decreasing IL-1β and TNF-α expression) (Lee et al., 2011; Zhu et al., 2013), anti-cancer (Kim et al., 2010; Sun et al., 2011; Lou et al., 2017; Maimaitili et al., 2017; Maxwell et al., 2017), and neuroprotection (Jang et al., 2002; Song J. et al., 2016). In addition, systematic pharmacological studies have been performed in multi-species (He et al., 2013; Gao et al., 2014; Li et al., 2017).

Nevertheless, systematic evaluations of *Arctigenin's* toxicological characteristics are limited. In the present study, the possible dose-time-dependent sub-chronic toxicities in rodents are investigated.

## Materials and methods

### Chemicals and reagents

*Arctigenin* (C_21_H_24_O_6_, MW = 372.41) was supplied by the State Key Laboratory of Generic Manufacture Technology of Chinese Traditional Medicine (Lunan Pharmaceutical Group Co. Ltd, Linyi, Shan Dong province, China). *Arctigenin* solution was prepared by 0.5% carboxymethyl cellulose (CMC-Na, Anhui SUNHERE Pharmaceutical Excipients CO., LTD., Huainan, An Hui province, China). Pentobarbital sodium was obtained from Sigma (Sigma–Aldrich, St Louis, MO, United States). Rosuvastatin (internal standard) was obtained from Sigma (Sigma–Aldrich, St Louis, MO, United States). Methanol, formic acid and ammonium formate (chromatographic pure) were purchased from Fisher Scientific Co. (Fisher Scientific, Houston, TX, United States). A Milli-QR system was used to prepare Ultrapure HPLC-grade water (Millipore, Milford, MA, United States).

### Concentration, stability, and homogeneity of *arctigenin* solutions

The concentration, stability and homogeneity of the *Arctigenin* solutions were determined by HPLC (Agilent-1200, AGILENT, Beijing, China). For concentration determination, the upper, middle, and lower solutions were conducted in triplicate, respectively. The RSD (%) between the measured concentrations and theoretical values should be from 85 to 115%. For stability determination, the upper, middle, and lower *Arctigenin* solutions (prepared for 52 h) were detected in triplicate, respectively. The RSD (%) between the measured and theoretical peaks of the solution should be from 85 to 115%. For stability determination, the upper, middle, and lower *Arctigenin* solutions (prepared for 52 h) were detected in triplicate, respectively. The impurity peak ratio (AUC solution/AUC standard) should not be >0.5.

### Ethics statement

The experimental protocols were approved by the Animal Ethics Committee of Lunan Pharmaceutical Group Co. Ltd. And the investigational procedures adopted in this study were in accordance with the Guide for the Care and Use of Laboratory Animals [National Research Council (US) Committee for the Update of the Guide for the Care and Use of Laboratory Animals, 2011].

### Animals, grouping, and drug administration

Overall, 180 SD rats (90 females and 90 males, 6–8 weeks old) were supplied by Charles River Laboratories (Beijing Vital River Laboratory Animal Technology Co., Ltd., Beijing, China). The animals were feeded in GLP center (Good Laboratory Practice), which acceptance by the China Food and Drug Administration (CFDA), and supplied with standard rodent diet (SPF grade, supplied by Beijing Keao Xieli Feed Co., Ltd., Beijing, China) and water *ad libitum*, acclimatized to a controlled temperature (23 ± 2°C) and maintained under a 12/12-hlight/dark cycle. The cage beddings and water bottles were cleaned on a daily basis. The animals were allowed 2 weeks of acclimatization before the commencement of experimental procedures. The standard of feeding environment is based on People's Republic of China national standard (GB14925-2010) and the feeding environment control system is SIMATIC S7-300 automatic control system. The rats were randomly divided into four groups, vehicle (30 rats), ARC-12 mg/kg (50 rats), ARC-36 mg/kg (50 rats), ARC-120 mg/kg (50 rats), 5 rats per cage. In the ARC-treated groups, 20 rats per group were used for the toxicokinetics study. When animals needed to be sacrificed, rodent ventilator was used to inhalation anesthesia.

### General observation

Cage-side examinations for apparent signs (behavior, mental status, gland secretion, respiration status, feces charicters genitals, and death) of toxicity or injury were conducted once a day after daily drug exposure, and once experimental animals develop toxic symptoms, increase observation frequency. And during the recovery period, the observation performed once a day. The standard of general observation were in accordance with the Guide for the Care and Use of Laboratory Animals published by the National Academies Press (Ed 8) (National Research Council, 2011).

### Body weight and food consumption

The body weight in all groups were measured once before the first drug exposure and then measured twice per week (on Monday and Thursday) during the drug exposure period and then measured weekly (on Thursday) during the recovery period.

Food consumptions were detected weekly (on Sunday) during the dosing and recovery periods. On the days of the food consumption assays, 300 g animal feed was delivered, and the remaining portions were measured at the same time on the second day (on Monday), and the food consumption was calculated.

### Urine, hematologic, and biochemical examinations

Urine examinations were performed once at the end of the drug exposure (day 29th) and recovery periods (day 57th), respectively. The detail procedures were performed as previous described (Tian et al., 2017). In briefly, the animals were placed in metabolic cages individually, fasted but free access to water. Urine were collected for 16 h, processed and detected within 2 h. Urine parameters, including GLU, BIL, KET, SG, BLO, PH, PRO, URO, NIT, and WBC, were detected by a Urine Analyzer (CLINITEK STATUS, Erlangen, Germany).

At the end of the drug exposure (day 29th) and recovery periods (day 57th), inhalation anesthesia of animals were performed and abdominal aortic vein blood (4.9 ml) were collected. All animals were fasting for 12 h before sampling.

For the hematological cytology assay, 1 ml of blood was anticoagulated by EDTA. For clotting time detect, 0.9 ml of blood was anticoagulated by sodium citrate (1,800 *g*) for 10 min at room temperature, and the plasma was separated for examination. Hematological parameters including RBC, HGB, HCT, MCV, MCH, MCHC, RET %, WBC, NEU, LYM, MONO, EOS, BASO, PLT, PT, and APTT were detected by an Automatic Five Classification Blood Analyzer (Cell-Dyn 3700, ABBOTT, United States) and Automatic Hemagglutination Analyzer (MC-4000, TECO, Germany), respectively.

For the biochemical examinations, 3 ml blood was centrifuged (1,800 *g*, 10 min, RT). Serum was prepared for the examinations. Parameters including AST, ALT, CPK, CK-MB, ALP, LDH, GGT, UREA, TP, ALB, A/G, GLU, TBIL, CREA, CHOL, TG, AMY-P, LIPC, Ca, P, Na^+^, K^+^, and Cl^−^ were detected by an Automatic Blood Biochemical Analyzer (BS-200, MINDRAY, Shenzhen, China).

### Autopsy and histopathology examination

Autopsy was performed at the end of the drug exposure (the 29th day, 20 rats were autopsied per group) and recovery periods (the rest of the rats, 10 rats were autopsied per group). The brain, spleen, thymus, lung, heart, liver, testis (bilateral)/ovaries (bilateral), kidneys (bilateral), and adrenal glands (bilateral) were separated and weighed.

To detect tissue injury, the histopathology examination was performed as previous described (Akindele et al., 2014). In briefly, rat tissues were fixed in 4% paraformaldehyde buffer for 24 h, dehydrated in graded alcohol (70, 90, 95, and 100%) and embedded in paraffin. Subsequently, paraffin blocks were cut into 2-μm sections and then subjected to routine hematoxylin-eosin (H&E) staining according to previous protocols (Fei et al., 2017). The photomicroscopic assessment using BX53 photomicroscope (Olympus, Tokyo, Japan), and the histopathology slides were viewed at various magnifications (× 40, × 100, and × 400) to detect pathological lesions.

### Ophthalmic examination

At the end of drug exposure (the 29th day) and recovery (the 42nd day) periods, tropicamide was used for mydriasis, cornea, iris, crystalline lens, anterior/atria chamber, and fundus oculi examinations of all animals by a Binocular Indirect Ophthalmoscope (Cat. #YZ25A, 6-6 VISION TECH Co., Ltd., Suzhou, Jiangsu province, China).

### Toxicokinetics study

#### Selectivity and matrix effect

To determine the influence of endogenous interference, the chromatograms derived from blank plasma, blank plasma with adding internal standard (IS) and blank plasma with the addition of *Arctigenin* were compared with chromatograms of all samples spiked with *Arctigenin* and IS. The structures of *Arctigenin* and rosuvastatin (IS) are shown in Figure S1. The typical chromatograms of samples spiked with *Arctigenin* and IS are shown in Figure S2. Under the UPLC-MS/MS conditions described above, the retention time of *Arctigenin* was approximately 2.07 min, and no interference peak was detected during the retention times in the blank plasma, tissues, or even samples after *Arctigenin* consumption. The matrix effect values for *Arctigenin* at different levels were 99.7–102%, indicating that no significant matrix effect was observed in the plasma and tissue samples under the present experimental conditions.

#### Linearity and the lower limits of quantitation

A linear relationship was found between the peak area and *Arctigenin* concentrations within the concentration ranges of 10–1,000 μg/L. The coefficient of determination (R^2^) was >0.99. The lower limit of quantitation (LLOQ) was 10 μg/L for *Arctigenin* in plasma (data not shown).

#### Precision and accuracy

The precision and accuracy were assessed by evaluating quality control (QC) samples (*n* = 6) at three concentration levels. The accuracy values ranged from 1.80 to 4.20%, and the intra- and inter-day precisions were 1.90–5.70% and 4.30–10.40%, respectively (data not shown). These results indicated that the presented UPLC-MS/MS method was accurate, reliable, and reproducible.

#### Recovery and stability

The recoveries of *Arctigenin* at three different QC concentrations (*n* = 6) in plasma were 100 −103%, and the recovery of IS was 102% (data not shown). The samples were stable after being placed at room temperature for 1 h (RE was from−2.8 to−0.8%) or stored at −20°C for 30 days (RE was from −1.5 to −0.7%) or even subjected to freeze–thaw cycles (RE were from 0.7 to 0.9%). In addition, the treated samples were stable at 4°C in an autosampler for 24 h, and the RE ranged from −7.1 to −1.5%, which indicated that large-scale samples could be detected in each analytical run. Based on the above results, a reliable, reproducible, and robust method has been developed and validated.

#### Sample prepare

A total of 0.1 ml of the rats' jugular vein blood was obtained from 0 min to 5 h after ARC administration. The blood samples were centrifuged at 3,000 rpm for 10 min at 4°C to obtain the plasma (50 μl). After adding 10 μl of internal standard (IS, 1 μg/ml) and 90 μl of methanol, centrifuging at 10,000 rpm for 10 min at 4°C, and repeating this step once to obtain the supernatant, 100-μl samples were collected, and the ARC concentrations were detected by HPLC-MS/MS. Kinetic parameters (AUC_0−t_, C_max_, and T_max_) were calculated.

### Data analysis

Data were calculated and analyzed with Excel 2010 (Microsoft, Redmond, WA, USA) and SPSS V19.0 (SPSS, Inc., Chicago, IL, United States). Toxicokinetic parameters, including C_max_, T_max_, and AUC_0−∞_, and the compartment model were analyzed by the Drug and Statistics (DAS) 3.0 software (Chinese Mathematical Pharmacology Society, Beijing, China). All values were presented as the mean ± SD, using an unpaired *t*-test by SPSS Statistics V19.0.

## Results

### Body weight

**Figure 1:**
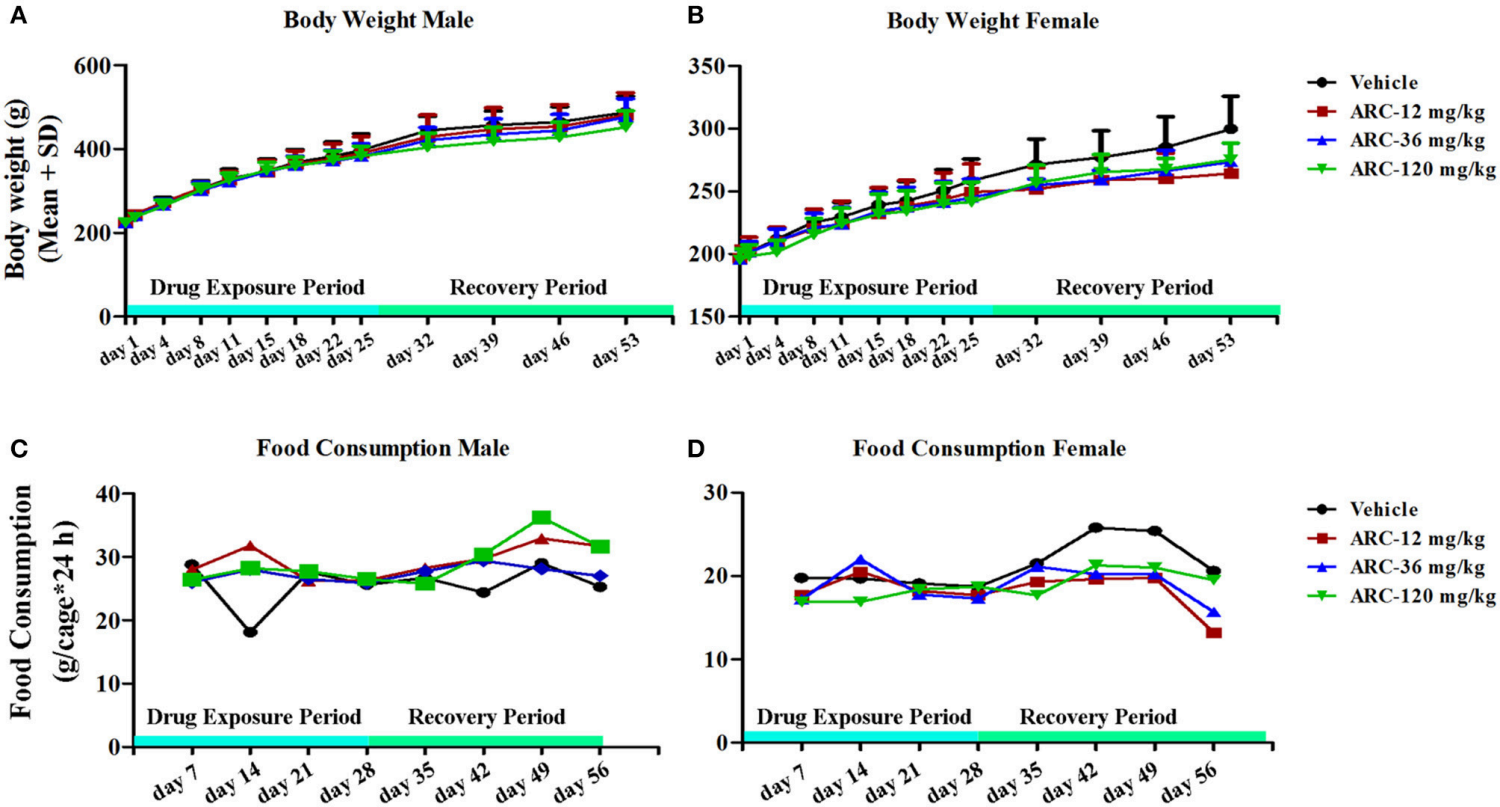
Body weight and food consumption of rats treated with *Arctigenin* at sub-chronic toxicity levels. **(A)** Body weight of males. **(B)** Body weight of females. **(C)** Food consumption of males. **(D)** Food consumption of females. (*n* = 10 or 5 per treatment group; results are presented as the mean + SD).

To investigate the influence of *Arctigenin* on body weight, all animals' body weights were detected before the first drug exposure (Day 0), drug exposure period (*n* = 30, 15 female and 15 male) and recovery period (*n* = 10, 5 female and 5 male). As shown in Table S1 and Figures 1A,B, compared with the control group, the body weight of the high dosage *Arctigenin* (ARC-120 mg/kg) group was significantly decreased (201.2 ± 9.7 vs. 211.5 ± 9.3, *P* < 0.05) at day 4. There were no differences in body weight in any group during the drug exposure and recovery periods.

### Food consumption

To further determine whether Arctigenin could influence the animals' ingestion, the food consumptions of the animals were detected in each week during the drug exposure and recovery periods. The results showed no differences in the food consumptions among the 4 groups (Table S2 and Figures 1C,D).

### Urine examination

Urine samples were collected and examined at the end of the drug exposure and recovery periods. As shown in Table 1, compared with the control group, there were no significant differences in any parameters including GLU, BIL, KET, SG, BLO, PH, PRO, URO, NIT, and WBC when rats were treated by *Arctigenin* (*P* > 0.05).

**Table 1:** The urine examination paremeters of *Arctigenin* (12-, 36-, and 120 mg/kg) administration by gavage (i.g) in rats during drug exposure period (*n* = 30, 15 female, 15 male) and recovery period.

**Parameters**	**Drug exposure period**								**Recovery period**							
	**Control**		**ARC-12 mg/kg**		**ARC-36 mg/kg**		**ARC-120 mg/kg**		**Control**		**ARC-12 mg/kg**		**ARC-36 mg/kg**		**ARC-120 mg/kg**	
	**Grade**	**NO**.	**Grade**	**NO**.	**Grade**	**NO**.	**Grade**	**NO**.	**Grade**	**NO**.	**Grade**	**NO**.	**Grade**	**NO**.	**Grade**	**NO**.
GLU	—	30	—	30	—	30	—	30	—	10	—	9	—	10	—	10
	TQ	0	TQ	0	TQ	0	TQ	0	TQ	0	TQ	1	TQ	0	TQ	0
BIL	—	30	—	30	—	29	—	30	—	9	—	9	—	10	—	9
	1+	0	1+	0	1+	1	1+	0	1+	1	1+	1	1+	0	1+	1
KET	—	16	—	17	—	14	—	18	—	9	—	9	—	10	—	9
	TQ	3	TQ	2	TQ	8	TQ	4	TQ	1	TQ	1	TQ	0	TQ	1
	1+	11	1+	8	1+	8	1+	8	1+	0	1+	0	1+	0	1+	0
S.G	≤1.005	0	≤1.005	3	≤1.005	1	≤1.005	0	≤1.005	0	≤1.005	0	≤1.005	0	≤1.005	0
	1.01	6	1.01	11	1.01	5	1.01	4	1.01	0	1.01	0	1.01	0	1.01	0
	1.015	22	1.015	10	1.015	17	1.015	19	1.015	5	1.015	7	1.015	6	1.015	5
	1.02	2	1.02	6	1.02	7	1.02	6	1.02	4	1.02	3	1.02	4	1.02	5
	1.025	0	1.025	0	1.025	0	1.025	1	1.025	1	1.025	0	1.025	0	1.025	0
BLD	—	22	—	22	—	21	—	23	—	9	—	10	—	7	—	8
	TQ-Lysed	1	TQ Lysed	1	TQ Lysed	5	TQ Lysed	3	TQ Lysed	0	TQ Lysed	0	TQ Lysed	2	TQ Lysed	0
	1+	7	1+	7	1+	3	1+	3	1+	1	1+	0	1+	1	1+	1
	2+	0	2+	0	2+	1	2+	1	2+	0	2+	0	2+	0	2+	1
PH	6.5	1	6.5	0	6.5	2	6.5	0	6.5	0	6.5	0	6.5	0	6.5	0
	7	4	7	7	7	6	7	5	7	0	7	0	7	0	7	0
	7.5	2	7.5	5	7.5	1	7.5	3	7.5	0	7.5	1	7.5	0	7.5	0
	8	5	8	6	8	4	8	3	8	0	8	1	8	1	8	1
	8.5	18	8.5	12	8.5	17	8.5	17	8.5	8	8.5	6	8.5	7	8.5	6
	≥9	0	≥9	0	≥9	0	≥9	2	≥9	2	≥9	2	≥9	2	≥9	3
PRO	—	14	—	16	—	9	—	6	—	1	—	0	—	2	—	1
	TQ	2	TQ	0	TQ	2	TQ	4	TQ	2	TQ	2	TQ	1	TQ	1
	1+	8	1+	4	1+	8	1+	9	1+	3	1+	6	1+	2	1+	3
	2+	6	2+	9	2+	10	2+	11	2+	4	2+	1	2+	5	2+	4
	3+	0	3+	1	3+	1	3+	0	3+	0	3+	1	3+	0	3+	1
URO	3.2	29	3.2	30	3.2	30	3.2	30	3.2	10	3.2	10	3.2	10	3.2	10
	16	0	16	0	16	0	16	0	16	0	16	0	16	0	16	0
	33	1	33	0	33	0	33	0	33	0	33	0	33	0	33	0
NIT	—	30	—	30	—	30	—	30	—	10	—	10	—	10	—	10
LEU	—	26	—	28	—	28	—	28	—	9	—	9	—	9	—	9
	TQ	4	TQ	2	TQ	2	TQ	2	TQ	1	TQ	1	TQ	1	TQ	1
Color	Yellow	30	Yellow	30	Yellow	30	Yellow	30	Yellow	10	Yellow	10	Yellow	10	Yellow	10

*TQ, Trace Quantity; BLD, blood; BIL, Bilirubin; URO, Urobilinogen; KET, Ketones; PRO, Protein; NIT, Nitrite; GLU, Glucose; S.G, Specific Gravity; LEU, Leucocytes; (n = 10, 5 female, 5 male per Treatment Group, Results Were Presented as Mean ± SD)*.

### Hematologic, biochemical examination

At the end of the drug exposure and recovery periods, the results of the hematologic examinations are shown in Table 2. The results demonstrated that compared with the control group, there were no significant differences in all hematologic parameters in the *Arctigenin*-treated groups (*P* > 0.05).

**Table 2:** The hematologic examination paremeters of *Arctigenin* (12-, 36-, and 120 mg/kg) administration by gavage (i.g) in rats' serum at the end of drug exposure period (*n* = 30, 15 female, 15 male) and recovery period (*n* = 10, 5 female, 5 male per treatment group, results were presented as Mean ± SD).

**Parameters**	**Drug exposure period**					**Recovery period**				
	**N0**	**Control**	**ARC-12 mg/kg**	**ARC-36 mg/kg**	**ARC-120 mg/kg**	**NO**.	**Control**	**ARC-12 mg/kg**	**ARC-36 mg/kg**	**ARC-120 mg/kg**
WBC	20	6.71 ± 2.60	5.95 ± 2.11	6.10 ± 2.28	6.27 ± 2.64	10	5.56 ± 2.98	5.16 ± 2.61	5.35 ± 3.71	4.93 ± 1.59
NEU	20	1.02 ± 0.67	0.87 ± 0.33	0.78 ± 0.26	0.93 ± 0.42	10	0.82 ± 0.56	0.95 ± 0.54	0.94 ± 0.51	0.83 ± 0.41
LYM	20	5.15 ± 2.10	4.59 ± 1.72	4.85 ± 1.97	4.77 ± 2.16	10	4.36 ± 2.29	3.77 ± 1.96	4.03 ± 3.08	3.68 ± 1.22
MONO	20	0.256 ± 0.163	0.245 ± 0.124	0.222 ± 0.108	0.270 ± 0.132	10	0.172 ± 0.117	0.212 ± 0.117	0.150 ± 0.106	0.198 ± 0.124
EOS	20	0.091 ± 0.037	0.079 ± 0.027	0.085 ± 0.033	0.092 ± 0.043	10	0.072 ± 0.027	0.072 ± 0.039	0.077 ± 0.042	0.078 ± 0.030
BASO	20	0.190 ± 0.101	0.167 ± 0.089	0.159 ± 0.093	0.200 ± 0.130	10	0.144 ± 0.097	0.159 ± 0.084	0.144 ± 0.087	0.151 ± 0.057
NEU %	20	14.8 ± 6.4	14.8 ± 3.2	14.0 ± 5.0	16.0 ± 5.8	10	14.6 ± 3.1	18.7 ± 6.3	19.2 ± 7.2	16.5 ± 5.6
LYM %	20	77.0 ± 7.9	76.9 ± 4.3	78.3 ± 5.6	74.9 ± 7.2	10	78.3 ± 4.2	72.2 ± 7.4	73.1 ± 8.5	74.8 ± 7.3
MONO %	20	3.79 ± 1.64	4.11 ± 1.60	3.69 ± 1.25	4.42 ± 1.46	10	3.10 ± 1.12	4.22 ± 1.07	3.28 ± 1.88	3.99 ± 1.78
EOS %	20	1.47 ± 0.60	1.47 ± 0.68	1.51 ± 0.62	1.58 ± 0.62	10	1.46 ± 0.57	1.72 ± 1.30	1.55 ± 0.54	1.64 ± 0.71
BASO %	20	2.86 ± 1.12	2.74 ± 0.91	2.52 ± 1.07	3.11 ± 1.26	10	2.49 ± 0.59	3.11 ± 0.83	2.81 ± 0.84	3.07 ± 0.75
RBC	20	7.16 ± 0.56	7.01 ± 0.53	6.95 ± 0.65	7.04 ± 0.58	10	7.13 ± 0.53	7.22 ± 0.41	7.46 ± 0.65	7.49 ± 0.39
HGB	20	136.2 ± 10.6	136.0 ± 8.2	135.7 ± 11.0	135.6 ± 11.0	10	133.7 ± 8.4	134.2 ± 5.3	135.6 ± 9.2	137.4 ± 3.8
HCT	20	47.8 ± 3.4	47.1 ± 3.1	46.8 ± 3.9	47.3 ± 4.2	10	46.5 ± 3.4	46.5 ± 1.4	47.2 ± 3.1	47.9 ± 1.6
MCV	20	66.9 ± 1.9	67.3 ± 1.5	67.5 ± 2.5	67.2 ± 2.2	10	65.2 ± 2.2	64.5 ± 2.7	63.4 ± 2.2	64.1 ± 2.1
MCH	20	19.1 ± 1.1	19.4 ± 0.5	19.5 ± 0.8	19.3 ± 0.7	10	18.8 ± 0.8	18.6 ± 0.9	18.2 ± 0.6	18.4 ± 0.8
MCHC	20	285.0 ± 14.7	288.9 ± 5.4	289.7 ± 4.4	286.8 ± 4.5	10	287.8 ± 6.9	288.4 ± 4.9	287.2 ± 3.8	286.3 ± 5.0
RDW	20	14.5 ± 1.0	14.7 ± 1.1	14.3 ± 1.1	14.6 ± 0.8	10	15.0 ± 0.9	14.7 ± 1.2	15.1 ± 1.0	15.1 ± 1.3
PLT	20	1,008.8 ±128.9	1,011.7 ± 98.2	1,009.7 ± 123.0	1,030.3 ± 101.5	10	919.4 ± 69.8	922.0 ± 96.3	958.2 ± 75.4	936.7 ± 111.5
RET %	20	7.132 ± 2.973	7.031 ± 2.904	7.854 ± 2.503	6.616 ± 2.767	10	8.078 ± 2.755	8.584 ± 3.018	7.461 ± 1.823	7.055 ± 1.614
RETICABS	20	504.9 ± 200.4	486.0 ± 189.7	538.9 ± 165.0	455.4 ± 169.6	10	572.4 ± 154.6	624.9 ± 235.7	557.2 ± 150.8	528.6 ± 127.1

*WBC, White Blood Cell Count; NEU, Neutrophil; LYM, Lymphocyte; MONO, Monocyte; EOS, Eosinophils; BASO, Basophil; RBC, Red Blood Cell Count; HGB, Hemoglobin; HCT, Haematocrit; MCV, Mean Corpuscular Volume; MCH, Mean Corpuscular Hemoglobin; MCHC, Mean Corpuscular Hemoglobin Concentration; PLT, Platelet; RET %, Reticulocyte (%); RETIC.ABS, Absolute reticulocyte count*.

The biochemical assay showed that at the end of the recovery period, compared with the control group (37.8 ± 4.2 μmol/L), CREA in the *Arctigenin*-12 mg/kg group (42.2 ± 6.1 μmol/L) was significantly increased (*P* < 0.05); however, this change was within normal ranges. Further, in neither the drug exposure period nor the recovery period were there any differences in the biochemical parameters in all *Arctigenin*-treated groups (Table 3).

**Table 3:** The biochemical examination paremeters of *Arctigenin* (12-, 36-, and 120 mg/kg) administration by gavage (i.g) in rats' serum at the end of drug exposure period and recovery period (*n* = 10, 5 female, 5 male per treatment group, results were presented as Mean ± SD).

	**Drug exposure period (Day 28)**					**Recovery period (Day 28)**			
**Parameters**	**Units**	**Control**	**ARC-12 mg/kg**	**ARC-36 mg/kg**	**ARC-120 mg/kg**	**Control**	**ARC-12 mg/kg**	**ARC-36 mg/kg**	**ARC-120 mg/kg**
		***N***** = 20**	***N***** = 20**	***N***** = 20**	***N***** = 20**	***N*** = **10**	***N*** = **10**	***N*** = **10**	***N*** = **10**
ALT	U/L	36.6 ± 7.4	37.4 ± 8.2	35.2 ± 8.4	36.2 ± 6.6	32.9 ± 7.3	36.5 ± 12.2	39.2 ± 19.3	34.8 ± 4.7
AST	U/L	115.6 ± 22.6	104.9 ± 25.1	108.4 ± 19.8	109.7 ± 24.0	94.4 ± 26.8	100.7 ± 49.4	104.6 ± 34.9	88.0 ± 24.2
ALP	U/L	156.3 ± 52.8	157.8 ± 61.3	156.5 ± 59.5	156.5 ± 49.7	99.7 ± 27.0	98.3 ± 34.3	105.6 ± 42.6	106.9 ± 39.3
GGT	U/L	1.8 ± 0.9	1.8 ± 0.3	2.0 ± 0.8	1.9 ± 0.8	1.2 ± 1.1	1.6 ± 0.5	2.0 ± 2.5	1.4 ± 0.7
T-Bil	μM	1.2 ± 1.0	1.5 ± 1.1	1.2 ± 0.9	1.5 ± 1.0	2.2 ± 0.9	2.2 ± 0.8	1.8 ± 0.9	2.0 ± 0.9
TP	g/L	54.9 ± 5.1	53.5 ± 3.4	54.4 ± 5.0	54.4 ± 3.6	55.1 ± 5.1	58.2 ± 6.9	57.0 ± 6.6	54.8 ± 5.8
ALB	g/L	33.4 ± 1.5	33.4 ± 1.3	33.4 ± 2.1	48.6 ± 66.9	34.0 ± 2.6	34.4 ± 2.7	34.6 ± 3.9	34.0 ± 2.8
A/G	—	1.58 ± 0.27	1.68 ± 0.18	1.61 ± 0.16	1.64 ± 0.15	1.64 ± 0.21	1.48 ± 0.23	1.54 ± 0.11	1.65 ± 0.14
UREA	mol/L	4.48 ± 0.73	4.78 ± 0.68	4.45 ± 0.47	4.60 ± 0.72	4.83 ± 0.58	4.67 ± 0.73	4.76 ± 0.78	5.01 ± 0.95
CREA	μM	40.6 ± 4.7	40.4 ± 5.0	39.9 ± 4.4	40.6 ± 4.4	37.8 ± 4.2	42.2 ± 6.1*	41.5 ± 2.6	36.9 ± 3.7
CPK	U/L	577.4 ± 243.8	470.2 ± 244.7	560.4 ± 191.7	539.7 ± 242.6	398.6 ± 238.5	394.9 ± 285.5	465.7 ± 278.5	390.4 ± 240.1
CHOL	mol/L	0.98 ± 0.17	0.96 ± 0.22	0.93 ± 0.25	1.40 ± 2.05	1.20 ± 0.11	1.10 ± 0.27	1.18 ± 0.22	1.11 ± 0.26
TG	mol/L	0.33 ± 0.16	0.39 ± 0.26	0.35 ± 0.25	0.31 ± 0.16	0.39 ± 0.17	0.41 ± 0.16	0.49 ± 0.35	0.41 ± 0.12
GLU	mol/L	6.67 ± 0.74	6.57 ± 0.55	6.60 ± 0.84	6.77 ± 0.76	7.11 ± 0.67	7.23 ± 0.82	7.28 ± 0.35	7.08 ± 0.46

* Compared with control group (C), P < 0.05.

*AST, Aspartate Aminotransferase; ALT, Aspartate Aminotransferase; ALP, Alkaline Phosphatase; GGT, γ-glutamyl transpeptidase; TBIL, Total Bilirubin; TP, Total Protein; ALB, Albumin; A/G, Albumin/Globulin; UREA, Ureum; CREA, Creatinine; CPK, Creatine Phosphokinase; CHOL, Cholesterol; TG, Triglyceride; GLU, Glucose; (n = 30, 15 female, 15 male)*.

The clotting time (PT and APTT) and electrolyte index (K^+^, Na^+^, Cl^−^) were not significantly different among the groups (*P* > 0.05, Table 4).

**Table 4:** The electrolyte paremeters of *Arctigenin* (12-, 36-, and 120 mg/kg) administration by gavage (i.g) in rats at the end of drug exposure period (*n* = 20, 10 female, 10 male) and recovery period (*n* = 10, 5 female, 5 male per treatment group, results were presented as Mean ± SD).

**Group**	**Drug exposure period**						**Recovery period**					
	**N0**.	**K^+^**	**Na^+^**	**Cl^−^**	**PT**	**APTT**	**N0**.	**K^+^**	**Na^+^**	**Cl^−^**	**PT**	**APTT**
		**mmol/L**	**mmol/L**	**mmol/L**	**Sec**.	**Sec**.		**mmol/L**	**mmol/L**	**mmol/L**	**Sec**.	**Sec**.
Control	20	4.30 ± 0.31	142.0 ± 2.2	110.5 ± 2.4	21.68 ± 1.22	16.29 ± 2.09	10	3.98 ± 0.27	146.2 ± 1.2	110.8 ± 1.7	21.1 ± 1.1	17.2 ± 2.6
ARC-12 mg/kg	20	4.02 ± 0.51	142.2 ± 2.8	110.6 ± 2.0	21.57 ± 1.22	16.77 ± 2.53	10	3.66 ± 0.50	145.3 ± 1.5	110.2 ± 1.9	21.1 ± 2.2	18.0 ± 3.7
ARC-36 mg/kg	20	4.09 ± 0.41	142.4 ± 3.1	110.7 ± 2.1	21.57 ± 1.22	16.77 ± 2.53	10	3.87 ± 0.41	145.9 ± 1.5	110.9 ± 1.1	21.2 ± 1.1	17.4 ± 3.1
ARC-120 mg/kg	20	4.01 ± 0.39	141.1 ± 1.9	109.6 ± 1.7	21.64 ± 2.34	16.12 ± 1.85	10	3.87 ± 0.36	145.7 ± 0.9	111.3 ± 1.1	21.3 ± 1.7	17.3 ± 2.6

*PT, Prothrombin Time; and APTT, Activated Partial Thromboplastin Time*.

### Autopsy and histopathology examination

#### General observations

It was demonstrated that there were no abnormalities at the end of the drug exposure period. However, autopsy found that one rat in the *Arctigenin*-36 mg/kg group (Male, NO. MM11) showed bilateral testis and epididymis atrophy, but the rest of the animals showed no obvious abnormalities. At the end of the recovery period, *Arctigenin*-12 mg/kg administration led to the weight of male rats' brain (2.2002 ± 0.0637 g) being higher than in the control group (2.0856 ± 0.0889 g); the details of viscera weights, viscera/body weights, and viscera/brain weights are shown in Figure S3 and Tables 5–7, respectively.

**Table 5:** Viscera weights of *Arctigenin* (12-, 36-, and 120-mg/kg) administration by gavage (i.g) in rats at the end of drug exposure (*n* = 20, 10 male, and 10 female) and recovery period (*n* = 10, 5 female, 5 male per treatment group, results were presented as Mean ± SD).

**Time**	**Organ**	**Female**					**Male**					
		**n**	**Control**	**ARC-12 mg/kg**	**ARC-36 mg/kg**	**ARC-120 mg/kg**	**Organ**	**n**	**Control**	**ARC-12 mg/kg**	**ARC-36 mg/kg**	**ARC-120 mg/kg**
DEP	Ovary	10	0.1796 ± 0.033	0.1598 ± 0.036	0.1780 ± 0.039	0.1758 ± 0.027	Testis	10	3.351 ± 0.4018	3.230 ± 0.2332	3.240 ± 0.2528	3.292 ± 0.1890
RP		5	0.1441 ± 0.023	0.1115 ± 0.022	0.1170 ± 0.020	0.1297 ± 0.027		5	3.536 ± 0.2469	3.6096 ± 0.173	3.0070 ± 0.839	3.4653 ± 0.275
DEP	Uterus	10	0.5336 ± 0.176	0.5381 ± 0.130	0.6775 ± 0.217	0.5291 ± 0.135	Epididymis	10	1.150 ± 0.0990	1.0525 ± 0.093	1.0422 ± 0.105	1.1096 ± 0.123
RP		5	0.7929 ± 0.251	0.7132 ± 0.225	0.5617 ± 0.103	0.6621 ± 0.178		5	1.369 ± 0.0921	1.3125 ± 0.129	1.2407 ± 0.234	1.3473 ± 0.100
DEP	Spleen	10	0.5864 ± 0.088	0.5820 ± 0.096	0.5991 ± 0.091	0.6051 ± 0.069	Spleen	10	0.722 ± 0.1523	0.7237 ± 0.130	0.6940 ± 0.076	0.7939 ± 0.075
RP		5	0.6636 ± 0.117	0.6074 ± 0.073	0.5339 ± 0.033	0.5019 ± 0.129		5	0.978 ± 0.1733	0.8498 ± 0.124	0.9064 ± 0.101	0.8656 ± 0.164
DEP	Liver	10	7.1052 ± 0.565	7.0049 ± 0.547	7.1058 ± 1.135	6.8258 ± 0.906	Liver	10	10.327 ± 1.662	10.4879 ± 0.77	10.0019 ± 1.46	10.5962 ± 1.83
RP		5	7.7999 ± 0.926	7.1107 ± 0.620	6.8821 ± 0.666	6.8375 ± 1.295		5	12.7917 ± 1.58	11.9834 ± 1.31	12.7912 ± 1.53	13.0084 ± 2.52
DEP	Adrenal	10	0.0773 ± 0.010	0.0754 ± 0.012	0.0787 ± 0.007	0.0757 ± 0.018	Adrenal	10	0.0605 ± 0.015	0.0673 ± 0.014	0.0595 ± 0.016	0.0617 ± 0.012
RP		5	0.0681 ± 0.019	0.0628 ± 0.011	0.0593 ± 0.007	0.0738 ± 0.016		5	0.0426 ± 0.005	0.0537 ± 0.012	0.0555 ± 0.016	0.0483 ± 0.012
DEP	Kidney	10	1.7350 ± 0.111	1.6145 ± 0.085	1.6429 ± 0.173	1.6857 ± 0.216	Kidney	10	2.7795 ± 0.258	2.8762 ± 0.225	2.6054 ± 0.209	2.6680 ± 0.356
RP		5	1.8097 ± 0.131	1.7450 ± 0.183	1.7551 ± 0.056	1.6911 ± 0.234		5	3.3293 ± 0.376	3.1854 ± 0.300	3.0464 ± 0.341	3.1222 ± 0.663
DEP	Thymus	10	0.5497 ± 0.206	0.4801 ± 0.065	0.5020 ± 0.093	0.5498 ± 0.069	Thymus	10	0.5567 ± 0.144	0.5335 ± 0.100	0.5336 ± 0.090	0.4979 ± 0.070
RP		5	0.4908 ± 0.150	0.3940 ± 0.070	0.3680 ± 0.101	0.3482 ± 0.070		5	0.4764 ± 0.038	0.4401 ± 0.073	0.5170 ± 0.109	0.5039 ± 0.071
DEP	Heart	10	0.9241 ± 0.086	0.8449 ± 0.110	0.8902 ± 0.097	0.8724 ± 0.076	Heart	10	1.2349 ± 0.177	1.2201 ± 0.124	1.2588 ± 0.145	1.2621 ± 0.143
RP		5	0.9937 ± 0.071	0.9905 ± 0.192	0.9183 ± 0.097	0.8690 ± 0.115		5	1.5629 ± 0.177	1.4958 ± 0.184	1.5194 ± 0.110	1.4627 ± 0.187
DEP	Lung	10	1.2057 ± 0.121	1.1803 ± 0.093	1.2323 ± 0.116	1.2375 ± 0.152	Lung	10	1.4233 ± 0.164	1.5642 ± 0.342	1.4485 ± 0.128	1.4841 ± 0.189
RP		5	1.3545 ± 0.145	1.2611 ± 0.060	1.1363 ± 0.199	1.1602 ± 0.086		5	1.6277 ± 0.147	1.5691 ± 0.374	1.5683 ± 0.133	1.6030 ± 0.180
DEP	Brain	10	1.9221 ± 0.072	1.8842 ± 0.060	1.9169 ± 0.104	1.9287 ± 0.081	Brain	10	2.0264 ± 0.106	1.9588 ± 0.120	2.0004 ± 0.138	2.0099 ± 0.250
RP		5	1.9091 ± 0.066	1.9512 ± 0.057	1.9735 ± 0.106	1.9205 ± 0.086		5	2.0856 ± 0.088	2.1007 ± 0.099	2.0262 ± 0.058	2.200 ± 0.063*

*DEP, Drug exposure period; RP, Recovery period*.

* *Compared with control group (C), P < 0.05*.

**Table 6:** Viscera/body weights of *Arctigenin* (12-, 36-, and 120-mg/kg) administration by gavage (i.g) in rats at the end of drug exposure (*n* = 20, 10 male and 10 female) and recovery period (*n* = 10, 5 female, 5 male per treatment group, results were presented as mean ± SD).

**Time**	**Organ**	**Female**					**Male**					
		**n**	**Control**	**ARC-12 mg/kg**	**ARC-36 mg/kg**	**ARC-120 mg/kg**	**Organ**	**n**	**Control**	**ARC-12 mg/kg**	**ARC-36 mg/kg**	**ARC-120 mg/kg**
DEP	Ovary	10	0.0934 ± 0.0165	0.0852 ± 0.0204	0.0932 ± 0.0221	0.0912 ± 0.0143	Testis	10	1.6536 ± 0.1793	1.6559 ± 0.1681	1.6236 ± 0.1273	1.6541 ± 0.1690
RP		5	0.0758 ± 0.0142	0.0571 ± 0.0115	0.0598 ± 0.0134	0.0673 ± 0.0124		5	1.7008 ± 0.1738	1.7208 ± 0.1037	1.4930 ± 0.4371	1.5759 ± 0.1300
DEP	Uterus	10	0.2782 ± 0.0935	0.2868 ± 0.0759	0.3549 ± 0.1166	0.2744 ± 0.0687	Epididymis	10	0.5678 ± 0.0444	0.5377 ± 0.0411	0.5233 ± 0.0638	0.5533 ± 0.0324
RP		5	0.4183 ± 0.1413	0.3681 ± 0.1246	0.2845 ± 0.0487	0.3431 ± 0.0821		5	0.6587 ± 0.0664	0.6251 ± 0.0592	0.6147 ± 0.1256	0.6118 ± 0.0285
DEP	Spleen	10	0.3048 ± 0.0419	0.3096 ± 0.0545	0.3127 ± 0.0464	0.3140 ± 0.0367	Spleen	10	0.3559 ± 0.0690	0.3695 ± 0.0615	0.3481 ± 0.0417	0.3994 ± 0.0535
RP		5	0.3486 ± 0.0661	0.3118 ± 0.0417	0.2711 ± 0.0203	0.2605 ± 0.0610		5	0.4685 ± 0.0766	0.4044 ± 0.0555	0.4475 ± 0.0511	0.3944 ± 0.0789
DEP	Liver	10	3.6960 ± 0.2531	3.7222 ± 0.3237	3.7154 ± 0.6355	3.5434 ± 0.4871	Liver	10	5.0914 ± 0.7254	5.3738 ± 0.5181	5.0187 ± 0.7710	5.2722 ± 0.6803
RP		5	4.0872 ± 0.4880	3.6463 ± 0.3321	3.4939 ± 0.3577	3.5666 ± 0.6914		5	6.1304 ± 0.6722	5.6974 ± 0.4841	6.3024 ± 0.5948	5.9089 ± 1.1062
DEP	Adrenal	10	0.0402 ± 0.0049	0.0401 ± 0.0069	0.0411 ± 0.0040	0.0391 ± 0.0088	Adrenal	10	0.0297 ± 0.0068	0.0346 ± 0.0079	0.0300 ± 0.0087	0.0311 ± 0.0073
RP		5	0.0356 ± 0.0099	0.0322 ± 0.0054	0.0300 ± 0.0030	0.0384 ± 0.0083		5	0.0204 ± 0.0018	0.0257 ± 0.0058	0.0273 ± 0.0075	0.0219 ± 0.0053
DEP	Kidney	10	0.9032 ± 0.0565	0.8572 ± 0.0435	0.8586 ± 0.1001	0.8762 ± 0.1249	Kidney	10	1.3736 ± 0.1320	1.4703 ± 0.1049	1.3076 ± 0.1299	1.3410 ± 0.2239
RP		5	0.9473 ± 0.0478	0.8950 ± 0.0991	0.8903 ± 0.0226	0.8814 ± 0.1218		5	1.5945 ± 0.1412	1.5148 ± 0.1030	1.5020 ± 0.1449	1.4179 ± 0.2913
DEP	Thymus	10	0.2858 ± 0.1046	0.2548 ± 0.0328	0.2632 ± 0.0556	0.2861 ± 0.0429	Thymus	10	0.2747 ± 0.0720	0.2732 ± 0.0535	0.2666 ± 0.0396	0.2489 ± 0.0329
RP		5	0.2576 ± 0.0812	0.2015 ± 0.0329	0.1883 ± 0.0597	0.1822 ± 0.0413		5	0.2288 ± 0.0215	0.2093 ± 0.0331	0.2560 ± 0.0579	0.2298 ± 0.0377
DEP	Heart	10	0.4807 ± 0.0415	0.4488 ± 0.0604	0.4662 ± 0.0641	0.4525 ± 0.0375	Heart	10	0.6089 ± 0.0768	0.6236 ± 0.0611	0.6305 ± 0.0695	0.6336 ± 0.0884
RP		5	0.5207 ± 0.0361	0.5090 ± 0.1081	0.4665 ± 0.0558	0.4534 ± 0.0651		5	0.7502 ± 0.0884	0.7106 ± 0.0658	0.7507 ± 0.0626	0.6649 ± 0.0834
DDP	Lung	10	0.6266 ± 0.0511	0.6265 ± 0.0460	0.6439 ± 0.0640	0.6413 ± 0.0703	Lung	10	0.7027 ± 0.0762	0.7980 ± 0.1584	0.7251 ± 0.0532	0.7530 ± 0.1496
RP		5	0.7103 ± 0.0813	0.6472 ± 0.0441	0.5784 ± 0.1106	0.6049 ± 0.0492		5	0.7834 ± 0.0993	0.7515 ± 0.1906	0.7734 ± 0.0505	0.7292 ± 0.0858

*DEP, Drug exposure period; RP, Recovery period*.

**Table 7:** Viscera/Brain weights of *Arctigenin* (12-, 36-, and 120-mg/kg) administration by gavage (i.g) in rats at the end of drug exposure (*n* = 20, 10 male and 10 female) and recovery period (*n* = 10, 5 female, 5 male per treatment group, results were presented as Mean ± SD).

**Time**	**Organ**	**Female**					**Male**					
		**n**	**Control**	**ARC-12 mg/kg**	**ARC-36 mg/kg**	**ARC-120 mg/kg**	**Organ**	**n**	**Control**	**ARC-12 mg/kg**	**ARC-36 mg/kg**	**ARC-120 mg/kg**
DEP	Ovary	10	0.1796 ± 0.033	0.1598 ± 0.036	0.1780 ± 0.039	0.1758 ± 0.027	Testis	10	3.351 ± 0.4018	3.230 ± 0.2332	3.240 ± 0.2528	3.292 ± 0.1890
RP		5	0.1441 ± 0.023	0.1115 ± 0.022	0.1170 ± 0.020	0.1297 ± 0.027		5	3.536 ± 0.2469	3.6096 ± 0.173	3.0070 ± 0.839	3.4653 ± 0.275
DEP	Uterus	10	0.5336 ± 0.176	0.5381 ± 0.130	0.6775 ± 0.217	0.5291 ± 0.135	Epididymis	10	1.150 ± 0.0990	1.0525 ± 0.093	1.0422 ± 0.105	1.1096 ± 0.123
RP		5	0.7929 ± 0.251	0.7132 ± 0.225	0.5617 ± 0.103	0.6621 ± 0.178		5	1.369 ± 0.0921	1.3125 ± 0.129	1.2407 ± 0.234	1.3473 ± 0.100
DEP	Spleen	10	0.5864 ± 0.088	0.5820 ± 0.096	0.5991 ± 0.091	0.6051 ± 0.069	Spleen	10	0.722 ± 0.1523	0.7237 ± 0.130	0.6940 ± 0.076	0.7939 ± 0.075
RP		5	0.6636 ± 0.117	0.6074 ± 0.073	0.5339 ± 0.033	0.5019 ± 0.129		5	0.978 ± 0.1733	0.8498 ± 0.124	0.9064 ± 0.101	0.8656 ± 0.164
DEP	Liver	10	7.1052 ± 0.565	7.0049 ± 0.547	7.1058 ± 1.135	6.8258 ± 0.906	Liver	10	10.327 ± 1.662	10.4879 ± 0.77	10.0019 ± 1.46	10.5962 ± 1.83
RP		5	7.7999 ± 0.926	7.1107 ± 0.620	6.8821 ± 0.666	6.8375 ± 1.295		5	12.7917 ± 1.58	11.9834 ± 1.31	12.7912 ± 1.53	13.0084 ± 2.52
DEP	Adrenal	10	0.0773 ± 0.010	0.0754 ± 0.012	0.0787 ± 0.007	0.0757 ± 0.018	Adrenal	10	0.0605 ± 0.015	0.0673 ± 0.014	0.0595 ± 0.016	0.0617 ± 0.012
RP		5	0.0681 ± 0.019	0.0628 ± 0.011	0.0593 ± 0.007	0.0738 ± 0.016		5	0.0426 ± 0.005	0.0537 ± 0.012	0.0555 ± 0.016	0.0483 ± 0.012
DEP	Kidney	10	1.7350 ± 0.111	1.6145 ± 0.085	1.6429 ± 0.173	1.6857 ± 0.216	Kidney	10	2.7795 ± 0.258	2.8762 ± 0.225	2.6054 ± 0.209	2.6680 ± 0.356
RP		5	1.8097 ± 0.131	1.7450 ± 0.183	1.7551 ± 0.056	1.6911 ± 0.234		5	3.3293 ± 0.376	3.1854 ± 0.300	3.0464 ± 0.341	3.1222 ± 0.663
DEP	Thymus	10	0.5497 ± 0.206	0.4801 ± 0.065	0.5020 ± 0.093	0.5498 ± 0.069	Thymus	10	0.5567 ± 0.144	0.5335 ± 0.100	0.5336 ± 0.090	0.4979 ± 0.070
RP		5	0.4908 ± 0.150	0.3940 ± 0.070	0.3680 ± 0.101	0.3482 ± 0.070		5	0.4764 ± 0.038	0.4401 ± 0.073	0.5170 ± 0.109	0.5039 ± 0.071
DEP	Heart	10	0.9241 ± 0.086	0.8449 ± 0.110	0.8902 ± 0.097	0.8724 ± 0.076	Heart	10	1.2349 ± 0.177	1.2201 ± 0.124	1.2588 ± 0.145	1.2621 ± 0.143
RP		5	0.9937 ± 0.071	0.9905 ± 0.192	0.9183 ± 0.097	0.8690 ± 0.115		5	1.5629 ± 0.177	1.4958 ± 0.184	1.5194 ± 0.110	1.4627 ± 0.187
DEP	Lung	10	1.2057 ± 0.121	1.1803 ± 0.093	1.2323 ± 0.116	1.2375 ± 0.152	Lung	10	1.4233 ± 0.164	1.5642 ± 0.342	1.4485 ± 0.128	1.4841 ± 0.189
RP		5	1.3545 ± 0.145	1.2611 ± 0.060	1.1363 ± 0.199	1.1602 ± 0.086		5	1.6277 ± 0.147	1.5691 ± 0.374	1.5683 ± 0.133	1.6030 ± 0.180
DEP	Brain	10	1.9221 ± 0.072	1.8842 ± 0.060	1.9169 ± 0.104	1.9287 ± 0.081	Brain	10	2.0264 ± 0.106	1.9588 ± 0.120	2.0004 ± 0.138	2.0099 ± 0.250
RP		5	1.9091 ± 0.066	1.9512 ± 0.057	1.9735 ± 0.106	1.9205 ± 0.086		5	2.0856 ± 0.088	2.1007 ± 0.099	2.0262 ± 0.058	2.200 ± 0.063*

*DEP, Drug exposure period; RP, Recovery period*.

#### Microscopic examination

**Figure 2:**
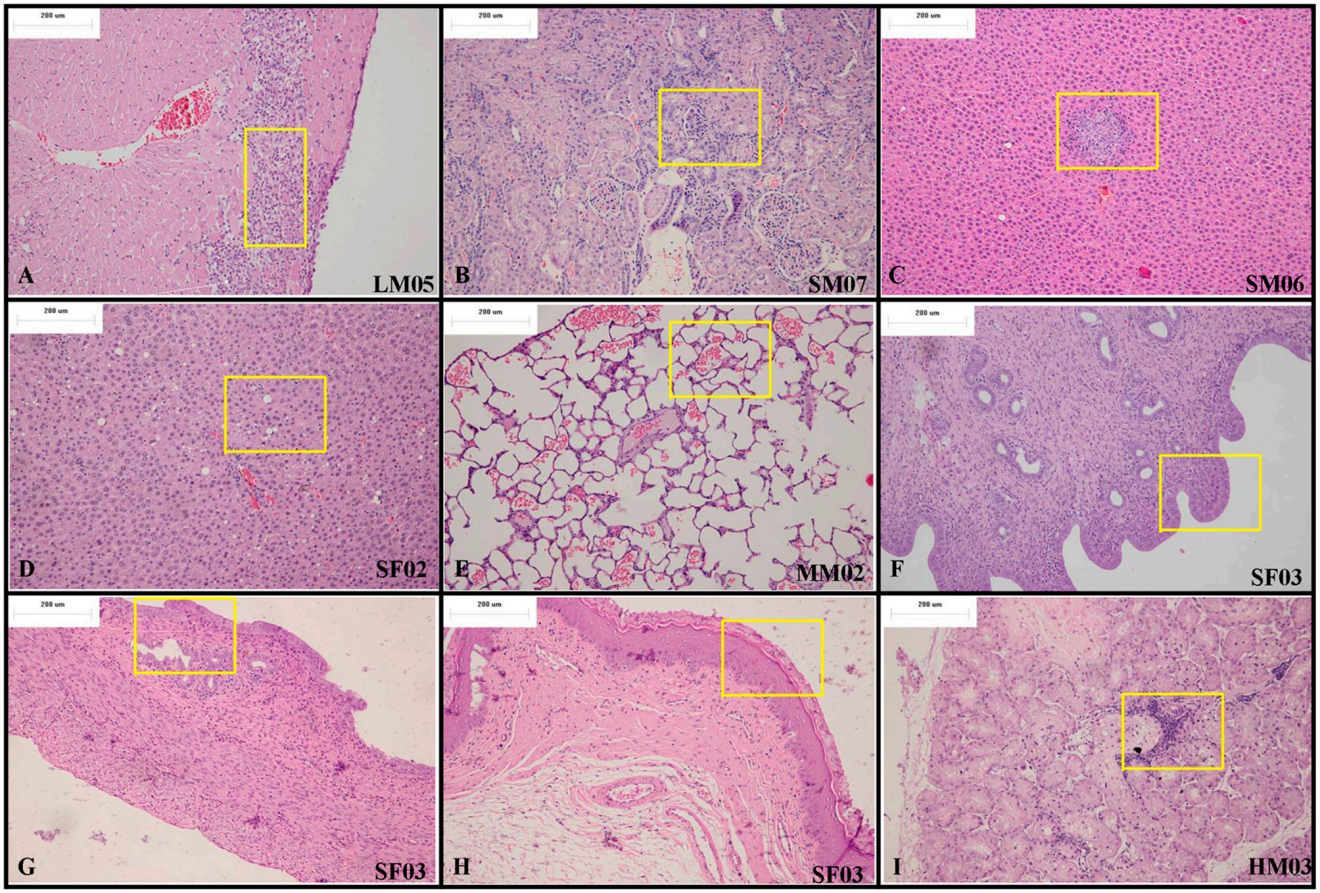
Representative histopathological results of rats at the end of the drug exposure period (Day 28). **(A)** In the apex of the heart, *Arctigenin*-12 mg/kg administration resulted in ventricular wall muscle cell interstitial lymphocyte infiltration. **(B)** In the kidney cortex, renal tubules are basophilic. In the liver, the hepatic lobular structure is complete, but there is partial hepatocyte focal necrosis and interstitial lymphocyte infiltration **(C)** and cytoplasmic vacuolation and fatty changes **(D)**. **(E)** In the lung and primary bronchus, erythrocyte and edema fluid were observed resulting from *Arctigenin*-36 mg/kg treatment. Changes of proestrus or estrus were observed in the uterus **(F)**, cervix **(G)**, and vagina intimal epithelial cells **(H)**, respectively. **(I)** Lymphocytic focal infiltration occurred in the prostate mesenchyme. (× 100 magnification).

At the end of the drug exposure period, H&E staining showed that *Arctigenin* could lead to multiple abnormal organs, including the heart, kidney, liver, lung (primary bronchus), spleen, uterus (cervix), vagina, prostate, submandibular gland, Hardwicke's gland, thyroid, parathyroid gland, and esophagus. The details of the abnormalities were as follows. In the apex of the heart, *Arctigenin*-12 mg/kg administration resulted in focal necrosis of ventricular septal muscle cells (Male, NO. LM03) and ventricular wall muscle cells (Male, NO. LM05) and interstitial lymphocyte infiltration (Figure 2A). In the kidney cortex, renal tubules were basophilic (HM03, SM07, Figure 2B), renal tubular epithelial cells were swollen (HM08, HM10), mineralized (HF07), and lymphocytes infiltrated (LF10). In the liver, the hepatic lobular structure was complete, but there was partial hepatocyte cytoplasmic vacuolation and fatty changes (SF02, MM02, LM05, Figure 2C), focal necrosis, and interstitial lymphocyte infiltration (SM06, MM01, MM04, LF10, Figure 2D). In the lung and primary bronchus, erythrocyte and edema fluid were observed in MM02 (Male), resulting from *Arctigenin*-36 mg/kg treatment (Figure 2E). Changes of proestrus (SF03, HF05, MF07, MF08, LF09) or estrus (MF01, MF04, LF02) were observed in the uterus, cervix, and vagina intimal epithelial cells, respectively (Figures 2F–H). Lymphocytic focal infiltration occurred in the prostate mesenchyme (SM02, SM04, HM04, MM01, and LM07, Figure 2I).

**Figure 3:**
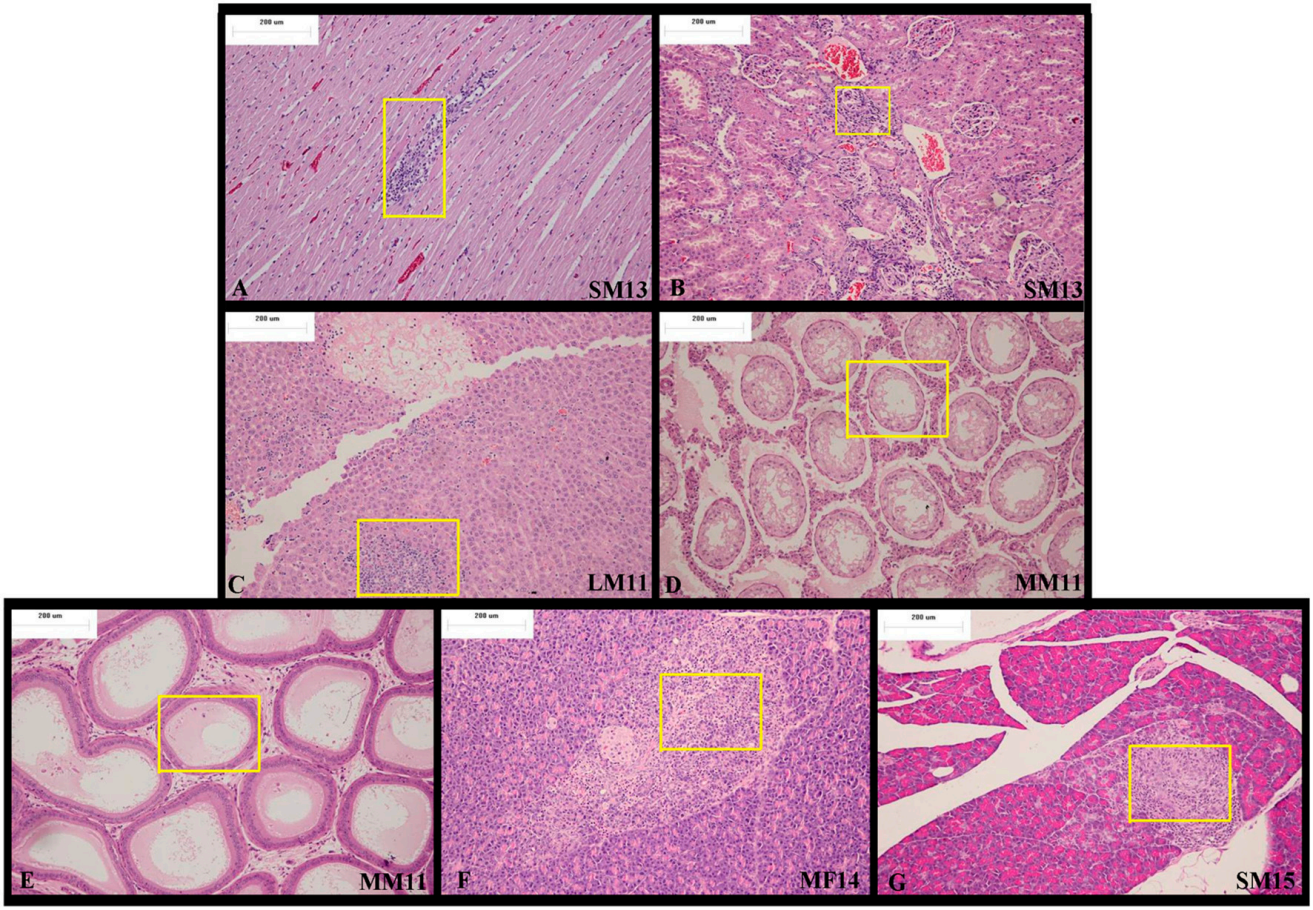
Representative histopathological results of rats at the end of the recovery period (Day 28). At the end of the recovery period, **(A)** in the apex of the heart, *Arctigenin* administration resulted in focal necrosis of ventricular wall muscle cells. **(B)** The interstitial substance of the renal cortex showed lymphocyte focal infiltration. **(C)** In the liver, the hepatic lobular structure is complete, but there is partial hepatocyte focal necrosis and interstitial lymphocyte infiltration. **(D–E)** Bilateral epididymis tube showed edema fluid, no spermatocytes, and interstitial chronic inflammatory cell infiltration. **(F–G)** In the pancreas exocrine gland, focal atrophy and interstitial lymphocytic infiltration were observed. (× 100 magnification).

At the end of recovery period, in the apex of the heart, *Arctigenin* administration resulted in focal necrosis of the ventricular septal muscle cells (Male, NO. LM14) and ventricular wall muscle cells (Male, NO.SM13, Figure 3A) and in interstitial lymphocyte infiltration. The interstitial substance of the renal cortex showed lymphocyte focal infiltration (SM13, HM11, HM12, HM13, HF13, LM12, and LM13, Figure 3B). In the liver, the hepatic lobular structure was integrated, but there were partial hepatocyte focal necrosis and interstitial lymphocyte infiltration (HF13, LM11, Figure 3C).

There was bilateral testicular fine tube atrophy, spermatozoic epithelium absence, mesenchyme hyperplasia, edema MM11 bilateral epididymis tube, edema fluid, no spermatocytes, accompanied with interstitial chronic inflammatory cell infiltration. (Figures 3D,E).

In the pancreas exocrine gland, focal atrophy and interstitial lymphocytic infiltration were observed in MF14 and SM15 (Figures 3F,G).

All histologic features of *Arctigenin* (12-, 36-, and 120 mg/kg) administration by gavage (i.g) in rats after drug exposure period and recovery period were showed in Table 8.

**Table 8:** The histologic features of *Arctigenin* (12-, 36-, and 120 mg/kg) administration by gavage (i.g) in rats after drug exposure period (*n* = 20, 10 female, 10 male) and recovery period (*n* = 20, 10 female, 10 male).

	**Histologic features**	**Drug exposure period (*****n*** = **20)**		**Recovery period (*****n*** = **20)**	
		**Animal number**	**Incidence**	**Animal number**	**Incidence**
Heart (Apex)	Focal necrosis (Ventricular septal myocytes) Lymphocytic infiltration (Interstitial)	LM03	5%	MM15	5%
	Focal necrosis (Left ventricular myocytes) Lymphocytic infiltration (Interstitial)	LMO5	5%	SM13	5%
	Focal necrosis (Papillary myocytes) Lymphocytic infiltration (Interstitial)	—	—	LM14	5%
Kidney	Basophilic change (Cortex)	SM07, LM08, HM03	15%	—	—
	Lymphocytic infiltration (Cortex)	LF10	5%	SM13, LM12, LM13, HM12, HM13, HF13	30%
	Cystic dilatation (Renal tubule)	HM08, HM10	10%	MM13	5%
Liver (Lobule)	Focal necrosis Lymphocytic infiltration	SM06, LF10, MM01, MM04	20%	LM11, LF14, HF13	15%
	Vacuolar and fatty degeneration (Cytoplasm)	SF02, LM05, MM02	15%	SM15, MF14	10%
Lung (Alveolus)	Red blood cells and edema fluid	MM02	5%	—	—
	Foam cells	—	—	LM12	5%
Testis	Proliferation, edema (Interstitial)	—	—	MM11	5%
Epididymis	Lymphocytic infiltration (Interstitial)	—	—	MM11	
Prostate	Lymphocytic infiltration (Interstitial)	SM02, SM04, LM07, MM01, HM04	25%	LM12, LM13, MM13, HM11, HM14	25%
Pancreas	Focal atrophy, Lymphocytic infiltration	—	—	SM15, MF14	10%
Haversian gland	Lymphocytic infiltration (Interstitial)	SM01, HM03	10%	SF15, HM14	10%
Esophagus	Focal necrosis, Lymphocytic infiltration (Interstitial)	MM09	5	—	—

*Animal number, the first letter means group (S, vehicle; L, low dose group, 12 mg/kg; M, middle dose group, 36 mg/kg; H, high dosa group, 120 mg/kg), the second letter means sex (M, male; F, female), the number means serial number*.

Autopsy and histopathology examinations demonstrated that the minimum dose of Arctigenin (12 mg/kg) resulted in adverse effects in several tissues and these effects were not ameliorated enve withdraw the drug exposure for 28 days. In contrast, although the primary adverse effects induced by Arctigenin (120 mg/kg) was lymphocytic infiltration, and involved in kidney cortex, liver lobule, and prostate, did not result in significant toxicity when consecutively given for 28 days or after recovery for 28 days. In our opinion, these un-dose-dependent and/or contradictory adverse effects induced by *Arctigenin* may be resulted from insufficient experimental period which hardly conclusive that whether these adverse effects will ameliorate or aggravate with the prolongation of drug exposure or recovery time.

### Rats' plasma toxicokinetics (TK) study

**Figure 4:**
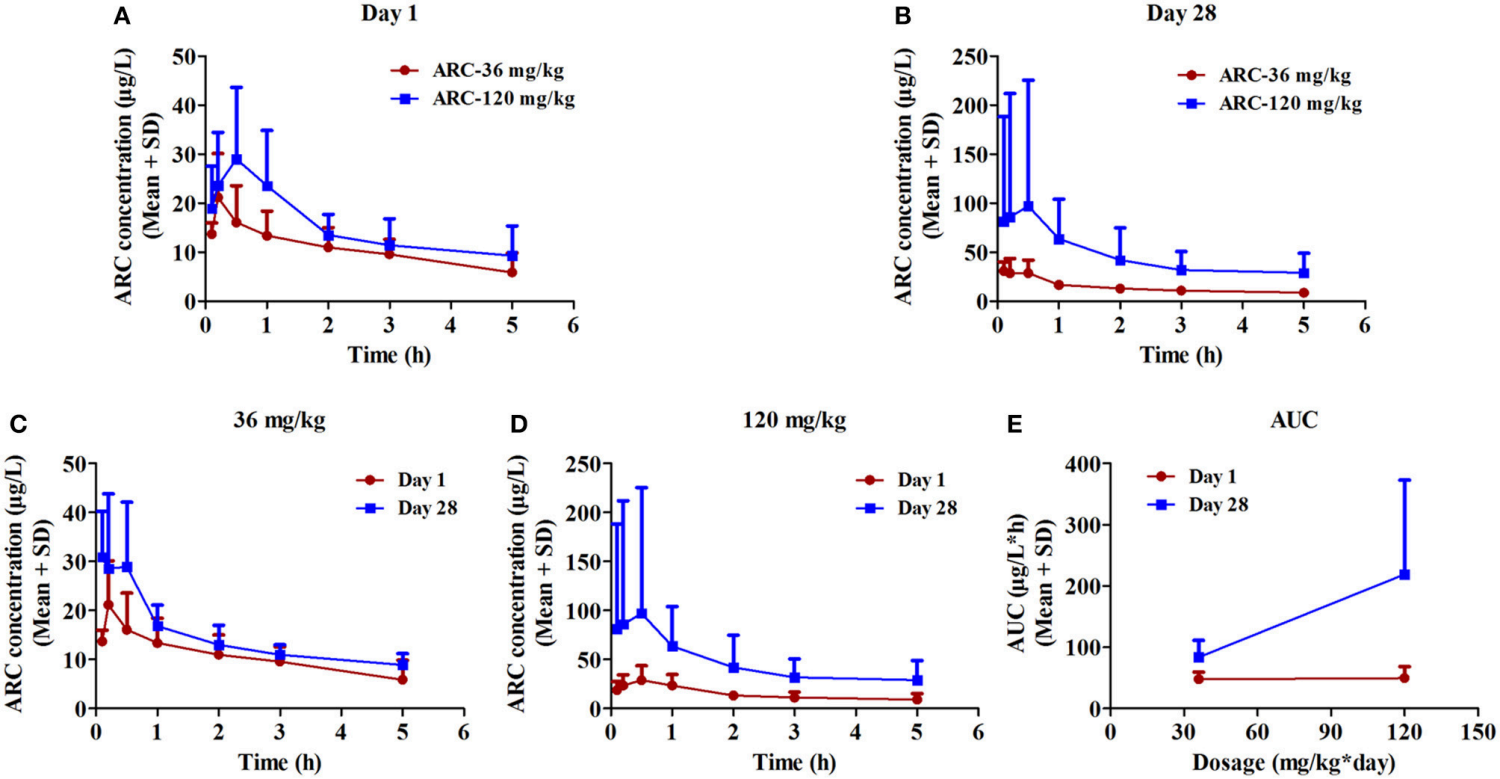
The plasma concentration vs. time profiles of *Arctigenin* in rats after the first **(A)** and last **(B)** intragastric administrations by *Arctigenin* at different doses. The plasma concentrations of *Arctigenin*in rats' plasma after i.g *Arctigenin* at the dosage of 36 mg/kg/day **(C)**, and 120 mg/kg/day **(D)**. **(E)** The exposed quantity of *Arctigenin*. (*n* = 10 in per treatment group; results are presented as the mean + SD).

To determine the toxicokinetic characteristics, the plasma concentration-time profiles of *Arctigenin* were detected following intragastric administration of 12-, 36-, and 120-mg/kg *Arctigenin* in rats (Figure 4), and the TK parameters are shown in Table 9. The results showed that the AUC_0−t_ ratio of males/females of the first drug exposure and last drug exposure ranged from 0.93 to 1.37. In female rats, the AUC_0−t_ was increased by 1.98-fold when the *Arctigenin* dosage increased from 36 to 120 mg/kg in first drug exposure, and this ratio reached to 4.11-fold at the last drug exposure. In contrast, the ratio of AUC_0−t_ (36 mg/kg)/ AUC_0−t_ (120 mg/kg) in male rats was 5.46-fold.

**Table 9:** Toxicokinetic parameters of *Arctigenin* (36- and 120 mg/kg) administration by gavage (i.g) in rats at the first drug exposure (day 1) and last drug exposure (day 28) (*n* = 10, 5 female, 5 male per treatment group, results were presented as Mean ± SD).

**Para**.	**Units**	**First drug exposure (Day 1)**		**Last drug exposure (Day 28)**	
		**ARC-36 mg/kg**	**ARC-120 mg/kg**	**ARC-36 mg/kg**	**ARC-120 mg/kg**
		**Mean ±SD**	**Mean ±SD**	**Mean ±SD**	**Mean ±SD**
AUC_0−t_	μg/L*h	48.3 ± 11.2	84.0 ± 27.1	49.7 ± 18.9	219 ± 154
AUC_0−∞_	μg/L*h	75.2 ± 23.5	198 ± 202	66.1 ± 32.3	495 ± 470
MRT_0−t_	h	1.71 ± 0.23	2.31 ± 0.47	1.39 ± 0.81	2.02 ± 0.49
	h	1.59 ± 0.56	4.74 ± 1.92	1.34 ± 0.66	5.35 ± 1.64
T_max_	h	0.2 ± 0.15	1.49 ± 2.00	0.2843 ± 0.172	1.2 ± 1.579
CL	L/h*kg	643 ± 153	1,020 ± 630	661 ± 294	520 ± 478
Vd	L/kg	1,040 ± 326	3,200 ± 1,390	1,110 ± 469	2,069 ± 1,436
Cmax	μg/L	29.6 ± 15.3	32.6 ± 12.2	40.7 ± 11.7	103 ± 27

In female rats, the ratio of AUC_day28_/AUC_day1_ (51.4 μg·L^−1^·h^−1^ and 48.3 μg·L^−1^·h^−1^ in the*Arctigenin*-36 mg/kg group, respectively) was 1.06 (below 2), which implied that the exposures were not different between the first and last drug exposure. However, the ratio of AUC_day28_/AUC_day1_ of female and male rats in the*Arctigenin*-120 mg/kg groups were 2.12 (female, 211 μg/L·h and 95.6 μg/L·h) and 3.26 (male, 227 and 69.7 μg/L·h), respectively. These results demonstrated that 120 mg/kg *Arctigenin*-administration could significantly increase the exposure.

When rats underwent intragastric administration (36 mg/kg), the *C*_*max*_were 29.6 ± 15.3 μg/L (Day 1) and 40.7 ± 11.7 μg/L (Day 28), and reached 32.6 ± 12.2 μg/L (Day 1) and 103 ± 27 μg/L (Day 28) with the 120 mg/kg dosage, respectively. The *T*_*max*_were 1.59 ± 0.56 h and 1.34 ± 0.66 h (36 mg/kg) and 4.74 ± 1.92 h and 5.35 ± 1.64 h (120 mg/kg), respectively.

## Discussion

Toxicity studies are considered vital components of herbal medicines' safety and provide evidence before further investigation in clinical trials. Although herbal extracts have been reported to possess multiple bioactivities and potential extensive applications (Gu et al., 2014; Chen et al., 2015; Song H. P. et al., 2016; Gao and Shen, 2017; Wu et al., 2017), the probable side effects are often neglected. For example, Arctigenin, the extract from *Arctium lappa* (burdock), a popular edible vegetable in China and Japan, is used as a general health tonic because of its multiple bioactivities *in vivo* and *in vitro* (Holetz et al., 2002; Liu et al., 2012; Fei et al., 2017), and this indiscriminate use lacks essential studies on its potential mild, moderate, and severe side effects and possible life-threatening effects. As we know, the potential toxicities of *Arctigenin* were reported previously. Repeated subcutaneous injection of large doses of *Arctigenin* can induce injury to the liver and biliary duct in Beagle dogs (Cai et al., 2018). Meanwhile, 7-day continuous oral exposure demonstrated that *Arctigenin* (9 mg/kg/day) evidently aggravated apoptosis in the kidney tissue, especially in tubular cells in kidney I/R mice, which implied that *Arctigenin* had potential renal injury effect (Han et al., 2018). However, the conclusions of these reports were either based on different animal models or different administrate routes, which unable to systematic evaluation the potential toxicities of continuous *Arctigenin* exposure. In the present study, the sub-chronic toxicity (28-day consecutive drug exposure and 28-day recovery) of *Arctigenin* in series concentrations (12, 36, and 120 mg/kg/day) were evaluated. Reversibility profiles, such as body weights, food consumptions, hematological, biochemical, histopathological, and toxicokinetical parameters, were evaluated in rats.

In the first week of the drug exposure period, compared with the control group, the body weight of the *Arctigenin*-120 mg/kg group was significantly decreased (201.2 ± 9.7 vs. 211.5 ± 9.3, *P* < 0.05), but this difference was eliminated following the subsequent drug exposure. However, the food consumptions were simultaneously detected, and the data showed that *Arctigenin* sub-chronic oral exposure with 12–120 mg/kg/day over the period of 28 days did not influence the food consumotion. These results suggest that *Arctigenin*-induced weight loss might be induced by either nutrient absorption inhibition or other unknown mechanisms.

With sub-chronic oral administration of 12–120 mg/kg/day of *Arctigenin* for 28 consecutive days, the hematological parameters such as cell counts (WBC, NEU, LYM, MONO, EOS, BASO, RBC), characteristics (HCT, MCV), components (HGB, MCH, MCHC, PLT, RET %) fluctuated in a non-dose dependent manner, and these alterations showed no significant differences. The hematopoietic system is considered the most sensitive targets for toxicities, soits index of physiological and pathological status are direct evidence for toxicology studies. Meanwhile, hematological parameters provide important information regarding bone marrow activity and intravascular status (anemia and hemolysis). The above results suggest that daily *Arctigenin* exposure at a dosage of 12–120 mg/kg for 28 continuous days showed no influence on hematological parameters.

To evaluate the influence of *Arctigenin* on liver functions, blood biochemical examinations were performed on day 28 of the drug exposure and recovery periods. Intragastric administration of *Arctigenin* at dosages of 12, 36, and 120 mg/kg in ratsdoes not significantly influence the liver enzymes (ALT, AST, GGT, and ALP) and other parameters such as proteins, cholesterol, and triglycerides in rats' serum. However, when rats are given 12 mg/kg/day *Arctigenin*, the serum CREA level is significantly increased compared with that ofthe control group (42.2 ± 6.1 vs. 37.8 ± 4.2 μM), but this increase was not observed in the 36 and 120 mg/kg/day group, which implies that *Arctigenin* did not deteriorate the liver function. As we know, the serum level of CREA is an indicator of kidney function, renal oxidative stress and inhibited NOS activity might cause increased CREA level and the change of renal histopathology, resulting in chronic renal damage (You et al., 2012). Our results revealed that 12 mg/kg *Arctigenin* induced an increase the serum CREA level as compared with control group, an elevation of its level in the blood is, thus, an indication of impaired kidney function, but this effect seems to be not dose-dependent since it wasn't detected in any of the higher dosage groups. Such a result may be caused by potential reasons. Firstly, serum CREA significantly increased were observed in two rats (LF11, 53.8, and LF13, 49.0 μM, respectively) by *Arctigenin*-12 mg/kg treatment. However, the histologic features of these two rats were not occurred obvious abnormality after recovery period, which may be because the injury degree was not serious enough to cause pathological changes. Secondly, as we known, the fluctuation range of SD rats' serum CREA is widely, and various among different study reports (Iga et al., 2010; Zhao et al., 2011; Cai et al., 2014). According to these results, although the CREA value (42.2 ± 6.1 μM) was increased in *Arctigenin* 12 mg/kg group, this difference within normal fluctuation range. The histopathological examination results show that variances with blood biochemical tests such as hepatocyte cytoplasmic vacuolation, fat change, focal necrosis, and interstitial lymphocyte infiltration of hepatocyte cytoplasm are observed in the control group, which means that the lesions are not serious enough to result in hepatic enzyme markers and other hepatic function parameters being significantly changed. As a synthetic and metabolic organ, the liver plays a vital role in vertebrates via its multidimensional physiological functions, such as blood supply, biliary flow, protein synthesis and secretion, detoxification of various metabolites, and production of biochemicals necessary for digestion. Any lesions caused by drugs, environmental chemicals, unhealthy life habits (such as smoking, alcoholism), and pathological status (such as obesity, diabetes) could interrupt the normal physiological functions. The serum ALT, AST, ALP, total protein and albumin levels are considered biomarkers for liver function. *Arctigenin* could not influence these liver enzyme markers, implying that *Arctigenin* is not hepatotoxic since the ALT, AST, and ALP levels are dramatically elevated and total protein, albumin levels are reduced under hepatotoxic conditions.

Urine examination and serum electrolytes could directly reflect renal function. The elevations of all of these parameters indicate renal injury or lesion. In the present study, *Arctigenin* treated animals' urine was collected and detected at the end of the drug exposure period (day 28) and recovery period (day 28), respectively. The results showed that neither nephritis/nephrolith marker (PRO and Leu), tubular function marker (S.G, PH, and GLU), and hemolytic marker (BIL and UBG) nor NIT and Ket were influenced in the *Arctigenin*-treated rats' urine. Meanwhile, the serum electrolytes such as sodium, potassium, and chloride levels showed no alterations when rats undergo 12–120 mg/kg/day *Arctigenin* for 28 days or even after withdrawal for 28 days. Although these results are contradictory with the histopathology results that showed that renal tubules are basophilic, the epithelial cells are swollen, mineralized, and lymphocyte infiltration, these uncommon lesions are insufficient to result in urine parameters and/or serum electrolytes alterations.

The toxicokinetic study demonstrated that *Arctigenin* was accumulated in organs when rats underwent 120 mg/kg exposure because of the ratio in this dosage was 2.60 (219 ± 154 μg/L·h vs. 84.0 ± 27.1 μg/L·h), which was no difference in 36-mg/kg treated groups (49.7 ± 18.9 μg/L·h vs. 48.3 ± 11.2 μg/L·h). In addition, organ accumulation of *Arctigenin* resulted in T_max_ significantly delated in 120 mg/kg group compared with 36 mg/kg (1.49 ± 2.00 h vs. 0.2 ± 0.15 h in day 1 and 1.2 ± 1.58 h vs. 0.28 ± 0.17 h in day 28, respectively). And that also explained that the half life (T_1/2_) of 36 mg/kg and 120 mg/kg groups showed significant difference. *Arctigenin* accumulated in stomach and liver resulted in prolonged the processes of absorption and elimination, respectively, which directly increased the Tmax and T1/2 (4.74 ± 1.92 h vs. 1.59 ± 0.56 h in day 1 and 5.35 ± 1.64 h vs. 1.34 ± 0.66 h in day 28, respectively) simultaneously. In additional, the significant variations of the plasma *Arctigenin* concentrations vs. time profiles especially during the first hour were observed in day 28, which may resulted from individual difference induced by one rat, HM19. Compared with other rats in the same group, the initial concentration (0.1 h) of *Arctigenin* in HM19 was 375 μg/L, but others were from 18.5 to 81.4 μg/L, and the high level of blood concentrations in HM19 were lasted until 2 h after drug exposure. In our opinion, these differences may be caused by either low elimination rate or high absorption rate of HM19. On one hand, low elimination rate resulted in drug accumulation in tissues and blood after continuous drug exposure, and on the other, high absorption rate after the last exposure caused drug absorbtion into the blood was rapidly. All these factors will result in significantly variations of the plasma *Arctigenin* concentrations.

All these conflicting results between the histopathological and blood biochemical/urine examinations might result from several reasons. First, inappropriate dose group. In our previously pharmacodynamic study of *Arctigenin in vivo*, the effective concentrations of *Arctigenin* were from 1 to 6 mg/kg, so we chosen 12 mg/kg (2 folds of the maximum effective dose-6 mg/kg) as the lowest dose in this study. Unfortunately, we have not observed effect in 12 mg/kg group. And the lowest dose will be adjusted in further study. Second, insufficient experimental period. 28-day continuous *Arctigenin* exposure and 28-day recovery period failed to provide more solid but conflicting evidences. Longer drug exposure and recovery period should be adopted in further study. Third, the spontaneously pathologic changes of experimental animals. According to previous studies, as the most commonly used experimental animals, SD rats may develpe some spontaneous diseases with the increase of feeding and result in pathologic changes in multiple organs or tissues (McInnes, 2011). In heart, focal inflammatory infiltration could be observed in different stage (from 6- to 32-weeks old). In kidney, mineralization could be observed in SD rats (incidence is 10% in 6-weeks old), and the incidence of Basophilic change (27-weeks), Lymphocytic infiltration (6-weeks) of cortex, cystic dilatation of renal tubule (6 weeks) were 5, 10, and 5%, respectively. In liver, the vacuolar degeneration is the most common spontaneous lesion in rats. The incidence of this pathologic change in 6-, 27-, and 31-weeks were 10, 25, and 10%, respectively. In addition, focal inflammatory infiltration (31-weeks) and necrosis (27-weeks) were both 5%. With the increase of age, different pathological changes including focal inflammatory infiltration (lung, 4-weeks of lung, 27-weeks of pancreas), atrophy (31-weeks of testis and prostate) will also obsvered in other tissues. According to these phenomena, together with almost all of the pathologic changes were simultaneously observed in control and *Arctigenin*-treated groups and cannot be ruled out because of spontaneously pathologic changes.

According to all results, the lowest obsvered adverse effect level (LOAEL) was induced by 12 mg/kg daily exposure to *Arctigenin*, and the No-observed-adverse-effect-level (NOAEL) should be lower than 12 mg/kg.

## Conclusion

In this study, sub-chronic toxicology studies of 28-day consecutive oral administration with 12–120 mg/kg/day of *Arctigenin* showed that that oral exposure to *Arctigenin* resulted in irreversible adverse effects in several tissues. Although these adverse effects were not simultaneously observed in the same animal, the conclusion of generally safe is inappropriate. This monomer extract from *Arctium lappa* (burdock) is a routine edible vegetable, which is associated with detrimental effects on the liver and renal functions of rats over a long-term period of drug exposure, and these toxic effects are irreversible even after withdrawal of the extract for 28 days. All these results suggest that daily ingestion of Arctium lappa should be performed with caution to avoid toxicities. Meanwhile, the development and application of *Arctigenin*, the active constituent of this herbal in the future should focus not only on its bioactivities but also on the potential toxicities, especially in the liver and kidney.

## Author contributions

JL and GZ contributed to the conception and design of the study and approved the final version to be submitted. YT, YR, and JL contributed to drafting the article. ZL and YR performed data analysis and interpretation. YT and LG performed the histopathological examination. LL, JY, and LC performed the blood and urine collection and detection. BL, XX, and JY performed the drug exposure, body weight and food consumption detection. MW, YL, and XL performed the sample detection for toxicokinetics assays.

### Conflict of interest statement

YT, YR, LG, LL, LC, BL, XL, JY, MW, YL, XX, JY, ZL, GZ, and JL were employed by Lunan Pharmaceutical Group Co., Ltd.

## Supplementary material

Figure S1 Representative the structures of *Arctigenin* and rosuvastatin (IS), and the ion-scan model by MS.

Figure S2 Representative extracted ion chromatograms of blank rats' plasma samples spiked with *Arctigenin*** (A)** and IS **(B)** (MRM model). Rats' blank plasma samples (with added *Arctigenin* and IS) spiked with AG **(C)** and IS **(D)** (MRM model). *Arctigenin* treated rats' plasma samples (with added IS) spiked with AG **(E)** and IS **(F)** (MRM model).

Figure S3 Viscera weights, viscera /body weights, and viscera /brain weights of rats treated with *Arctigenin* at sub-chronic toxicity levels. **(A)** Viscera weight of males. **(B)** Viscera weight of females. **(C)** Viscera/Body weight of males. **(D)** Viscera/Body weight of females. **(E)** Viscera/Brain weight of males. **(F)** Viscera/Brain weight of females (*n* = 10 or 5 per treatment group; results are presented as the mean + SD).

Table S1 The body weights (g) of *Arctigenin* (12-, 36-, and 120 mg/kg) administration by gavage (i.g) in rats during exposure period (*n* = 30, 15 female, 15 male) and recovery period (*n* = 10, 5 female, 5 male per treatment group, results were presented as Mean ± SD).

Table S2 The food consumption (g/cage^*^24 h) of *Arctigenin* (12-, 36-, and 120 mg/kg) administration by gavage (i.g) in rats during drug exposure period (*n* = 30, 15 female, 15 male) and recovery period (*n* = 10, 5 female, 5 male per treatment group, results were presented as Mean ± SD).

## Abbreviations

TQ: Trace Quantity
BLD: blood
BIL: Bilirubin
URO: Urobilinogen
KET: Ketones
PRO: Protein
NIT: Nitrite
GLU: Glucose
S.G: Specific Gravity
LEU: Leucocytes
WBC: White Blood Cell Count
NEU: Neutrophil
LYM: Lymphocyte
MONO: Monocyte
EOS: Eosinophils
BASO: Basophil
RBC: Red Blood Cell Count
HGB: Hemoglobin
HCT: Hematocrit
MCV: Mean Corpuscular Volume
MCH: Mean Corpuscular Hemoglobin
MCHC: Mean Corpuscular Hemoglobin Concentration
PLT: Platelet
RET %: Reticulocyte (%)
AST: Aspartate Aminotransferase
ALT: Aspartate Aminotransferase
ALP: Alkaline Phosphatase
GGT: γ-glutamyl transpeptidase
TBIL: Total Bilirubin
ALB: Albumin
A/G: Albumin/Globulin
UREA: Ureum
CREA: Creatinine
CPK: Creatine Phosphokinase
CHOL: Cholesterol
TG: Triglyceride
GLU: Glucose
PT and APTT: Prothrombin Time Activated Partial Thromboplastin Time.
