# Supplementary figures and images for: 28-Day Oral Chronic Toxicity Study of Arctigenin in Rats
Source: Front Pharmacol. 2018 Sep 26;9:1077. doi: 10.3389/fphar.2018.01077 (PMC6169246; doi:10.3389/fphar.2018.01077)

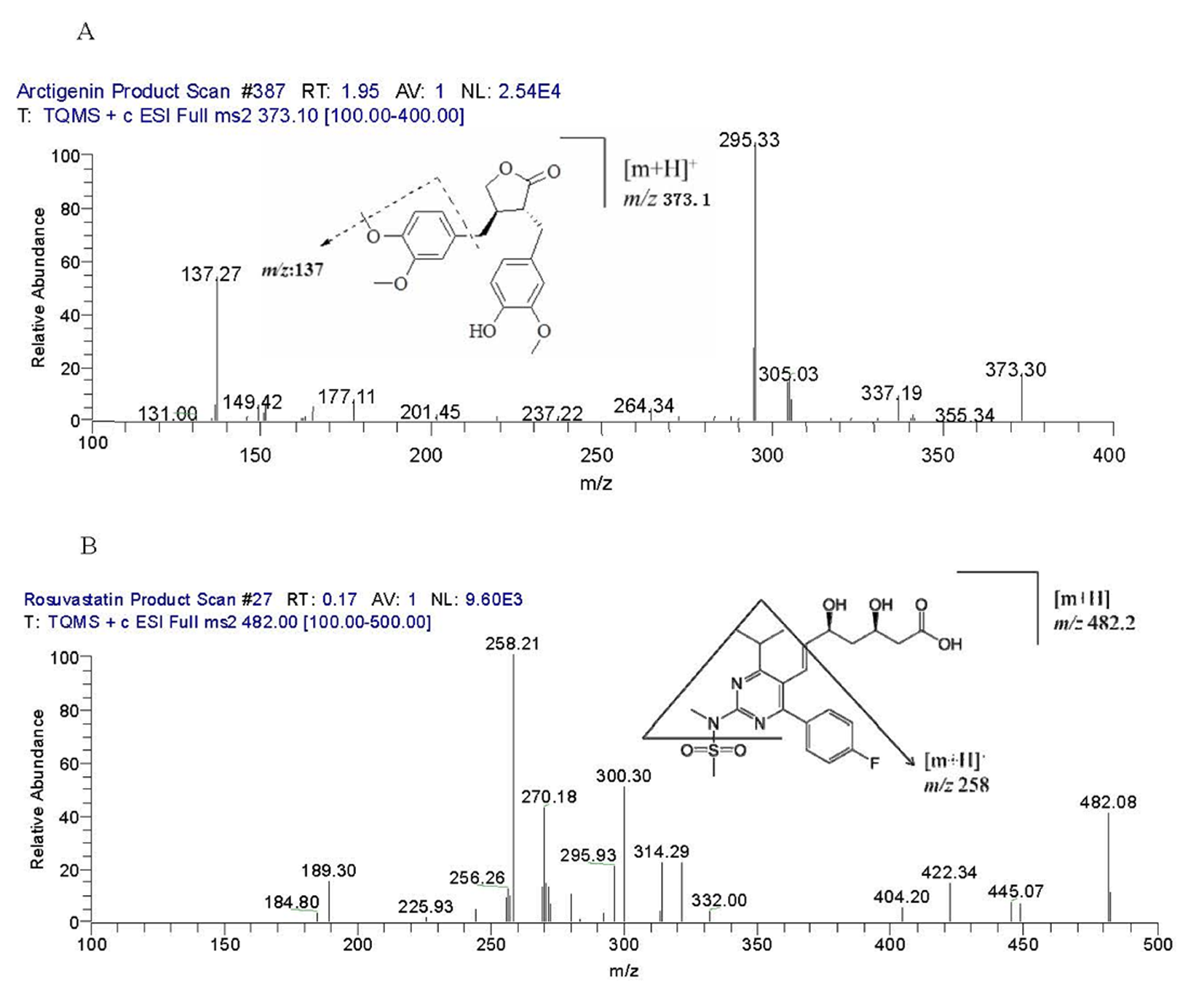

Supplement: Figure S1 — Representative the structures of Arctigenin and rosuvastatin (IS), and the ion-scan model by MS. [file Image_1.TIF]

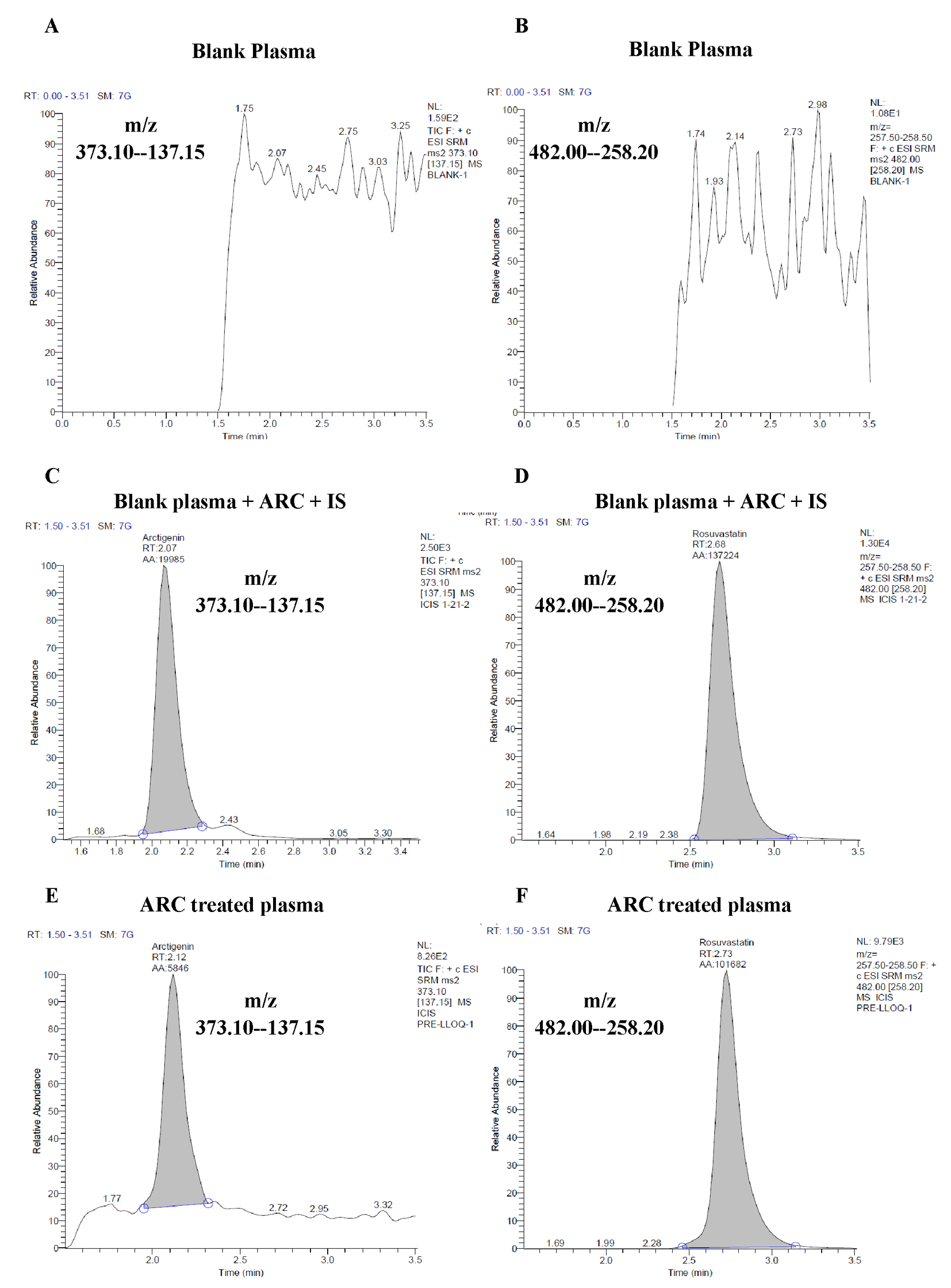

Supplement: Figure S2 — Representative extracted ion chromatograms of blank rats' plasma samples spiked with Arctigenin (A) and IS (B) (MRM model). Rats' blank plasma samples (with added Arctigenin and IS) spiked with AG (C) and IS (D) (MRM model). Arctigenin treated rats' plasma samples (with added IS) spiked with AG (E) and IS (F) (MRM model). [file Image_2.TIF]

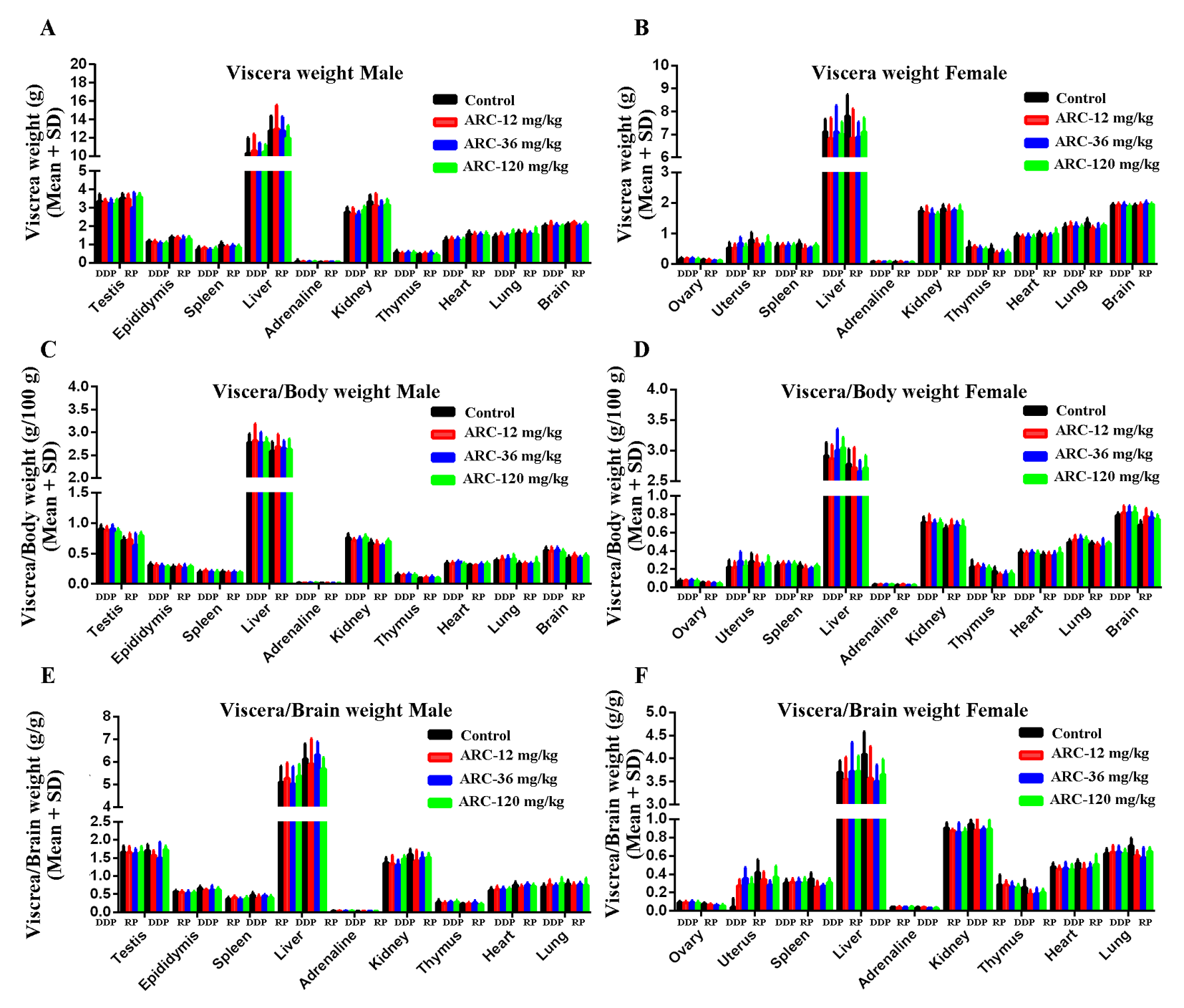

Supplement: Figure S3 — Viscera weights, viscera /body weights, and viscera /brain weights of rats treated with Arctigenin at sub-chronic toxicity levels. (A) Viscera weight of males. (B) Viscera weight of females. (C) Viscera/Body weight of males. (D) Viscera/Body weight of females. (E) Viscera/Brain weight of males. (F) Viscera/Brain weight of females (n = 10 or 5 per treatment group; results are presented as the mean + SD). [file Image_3.TIF]
