# Supplementary material for: 28-Day Oral Chronic Toxicity Study of Arctigenin in Rats
Source: Front Pharmacol. 2018 Sep 26;9:1077. doi: 10.3389/fphar.2018.01077 (PMC6169246; doi:10.3389/fphar.2018.01077)
Supplement: Table S1 — The body weights (g) of Arctigenin (12-, 36-, and 120 mg/kg) administration by gavage (i.g) in rats during exposure period (n = 30, 15 female, 15 male) and recovery period (n = 10, 5 female, 5 male per treatment group, results were presented as Mean ± SD). [file Table_1.DOCX]

**Table S1. The body weights (g) of Arctigenin (12-, 36-, and 120 mg/kg) Administration by Gavage (i.g) in rats During Exposure Period (n=30, 15 female, 15 male) and Recovery Period (n=10, 5 female, 5 male per Treatment Group, Results Were Presented as Mean ± SD).**

| **Group** | **Gender** | **N** |  | **Drug Exposure Period** | | | | | | | | **N** | **Recovery Period** | | | |
| --- | --- | --- | --- | --- | --- | --- | --- | --- | --- | --- | --- | --- | --- | --- | --- | --- |
|  |  |  | Day 0 | Day 1 | Day 4 | Day 8 | Day 11 | Day 15 | Day 18 | Day 22 | Day 25 |  | Day 32 | Day 39 | Day 46 | Day 53 |
| C | ♀ | 15 | 196.0±8.6 | 201.5±6.7 | 211.5±9.3 | 225.4±10.1 | 229.3±11.6 | 239.1±13.4 | 242.1±15.6 | 250.4±16.6 | 258.2±17.5 | 5 | 271.1±20.5 | 276.9±21.2 | 285.0±24.4 | 299.6±26.1 |
|  | ♂ | 15 | 224.6±9.6 | 238.2±12.2 | 272.1±12.7 | 305.5±19.1 | 329.0±22.8 | 346.3±30.0 | 368.5±30.7 | 382.0±34.9 | 397.2±39.1 | 5 | 444.0±34.0 | 457.2±33.6 | 464.9±35.9 | 487.7±37.9 |
| L | ♀ | 15 | 196.7±9.6 | 201.8±11.5 | 209.9±11.2 | 220.3±15.0 | 223.9±18.1 | 232.1±20.7 | 237.9±20.7 | 243.2±21.4 | 249.2±22.6 | 5 | 251.5±17.2 | 258.7±20.4 | 260.0±20.2 | 264.0±24.2 |
|  | ♂ | 15 | 225.4±10.2 | 243.6±7.3 | 272.9±10.7 | 306.3±15.3 | 325.5±21.3 | 345.9±27.2 | 364.4±31.7 | 376.3±36.1 | 390.4±38.6 | 5 | 428.5±52.5 | 447.2±51.3 | 453.8±52.4 | 481.1±53.2 |
| M | ♀ | 15 | 195.2±8.0 | 201.1±8.8 | 210.0±9.8 | 220.9±11.3 | 223.4±13.6 | 233.9±15.4 | 237.3±15.8 | 240.7±17.2 | 245.1±14.5 | 5 | 254.5±5.0 | 258.7±7.7 | 266.1±16.5 | 273.3±14.9 |
|  | ♂ | 15 | 223.9±9.0 | 240.5±7.5 | 266.0±14.9 | 300.5±20.3 | 321.1±20.4 | 345.6±23.3 | 361.0±22.8 | 370.7±27.0 | 382.5±30.1 | 5 | 421.2±30.2 | 434.8±37.4 | 444.9±38.2 | 476.8±43.4 |
| H | ♀ | 15 | 195.2±8.8 | 198.1±9.3 | 201.2±9.7* | 215.2±13.0 | 223.8±12.6 | 231.7±15.9 | 234.1±16.1 | 239.7±16.8 | 241.2±16.2 | 5 | 256.4±14.4 | 265.0±14.3 | 267.6±8.4 | 275.0±13.5 |
|  | ♂ | 15 | 223.8±10.5 | 237.8±7.3 | 264.0±14.4 | 302.6±14.2 | 326.0±16.9 | 349.3±19.4 | 360.4±20.8 | 373.0±22.9 | 383.4±23.9 | 5 | 403.7±34.4 | 417.8±33.7 | 427.9±36.2 | 452.2±39.0 |

*Compared with control group (C), *P* < 0.05.
