# Supplementary material for: 28-Day Oral Chronic Toxicity Study of Arctigenin in Rats
Source: Front Pharmacol. 2018 Sep 26;9:1077. doi: 10.3389/fphar.2018.01077 (PMC6169246; doi:10.3389/fphar.2018.01077)
Supplement: Table S2 — The food consumption (g/cage*24 h) of Arctigenin (12-, 36-, and 120 mg/kg) administration by gavage (i.g) in rats during drug exposure period (n = 30, 15 female, 15 male) and recovery period (n = 10, 5 female, 5 male per treatment group, results were presented as Mean ± SD). [file Table_2.DOCX]

**Table S2. The food consumption (g/cage*24 h) of Arctigenin (12-, 36-, and 120 mg/kg) Administration by Gavage (i.g) in rats During Drug Exposure period (n=30, 15 female, 15 male) and Recovery Period (n=10, 5 female, 5 male per Treatment Group, Results Were Presented as Mean ± SD).**

| **Group** | **Gender** | **Drug Exposure Period** | | | | **Recovery Period** | | | |
| --- | --- | --- | --- | --- | --- | --- | --- | --- | --- |
|  |  | Day 7 | Day 14 | Day 21 | Day 28 | Day 35 | Day 42 | Day 49 | Day 56 |
| C | ♂ | 28.8 | 18.1 | 27.6 | 25.6 | 26.7 | 24.4 | 29.0 | 25.3 |
|  | ♀ | 19.8 | 19.7 | 19.1 | 18.7 | 21.5 | 25.8 | 25.4 | 20.6 |
| L | ♂ | 28.0 | 31.8 | 26.2 | 26.3 | 28.3 | 29.7 | 32.9 | 31.7 |
|  | ♀ | 17.7 | 20.5 | 18.2 | 17.7 | 19.3 | 19.6 | 19.8 | 13.2 |
| M | ♂ | 26.0 | 28.0 | 26.5 | 25.9 | 27.7 | 29.4 | 28.1 | 27.0 |
|  | ♀ | 17.2 | 22.0 | 17.8 | 17.3 | 21.1 | 20.2 | 20.2 | 15.7 |
| H | ♂ | 26.4 | 28.2 | 27.7 | 26.5 | 25.8 | 30.4 | 36.2 | 31.6 |
|  | ♀ | 16.9 | 16.9 | 18.4 | 18.7 | 17.7 | 21.3 | 21.0 | 19.5 |

C, Control; L, Arctigenin-12 mg/kg; M, Arctigenin-36 mg/kg; H, Arctigenin-120 mg/kg.
